# Filamentous actin destabilization by H_2_O_2_ favors DnmA aggregation, with crucial roles of cysteines 450 and 776 in mitochondrial and peroxisomal division in *Aspergillus nidulans*

**DOI:** 10.1128/mbio.02822-23

**Published:** 2023-11-28

**Authors:** Verónica Garrido-Bazán, Dulce C. Guzmán-Ocampo, Laura Domínguez, Jesús Aguirre

**Affiliations:** 1 Instituto de Fisiología Celular, Departamento de Biología Celular y Desarrollo, Universidad Nacional Autónoma de México, Ciudad de México, Mexico; 2 Facultad de Química, Departamento de Fisicoquímica, Universidad Nacional Autónoma de México, Ciudad de México, Mexico; Universidad de Cordoba, Cordoba, Spain

**Keywords:** ROS signaling, mitochondrial dynamics, actin dynamics, dynamins, Drp1, fungal cell biology

## Abstract

The dynamin-like protein DnmA and its receptor FisA are essential for mitochondrial division in the fungus *Aspergillus nidulans*. Our research has demonstrated that H_2_O_2_ triggers significant changes in mitochondrial morphology, including constriction, division, and remodeling of the outer membrane. Here, we used genetics and confocal microscopy approaches to examine the impact of H_2_O_2_ on the cellular distribution of DnmA. Additionally, we evaluated the roles of conserved cysteines in mitochondrial and peroxisomal division. We show that H_2_O_2_ induces a DnmA aggregation consistent with higher-order oligomerization and its recruitment to mitochondria. This response is partially attributed to a rapid depolymerization and reorganization of actin caused by H_2_O_2_, implying that actin dynamics is also regulated by redox mechanisms. C295S, C380S, and C462S substitutions have minimal or no impact on DnmA function. In contrast, the C450S and C776S substitutions severely impair mitochondrial and peroxisomal division, despite not notably affecting the general distribution of DnmA and having opposite effects on DnmA oligomerization in the absence of FisA. Molecular dynamics simulations show that C450S and C776S substitutions as well as C450 oxidation have important effects on DnmA bundle signaling element-stalk domain angle, solvent-accessible surface area, and salt bridge interactions. The high probability of C450 oxidation and its associated changes in DnmA structure indicate that C450 oxidation by H_2_O_2_ would impact DnmA multimeric structure. We propose that H_2_O_2_ regulates mitochondrial division by orchestrating the generation of mitochondrial constrictions and the oligomerization of DnmA. In this model, DnmA C450 oxidation would constitute a necessary priming event for transition to the productive self-assembly required for mitochondrial scission.

**IMPORTANCE:**

Mitochondria constitute major sources of H_2_O_2_ and other reactive oxygen species in eukaryotic cells. The division of these organelles is crucial for multiple processes in cell biology and relies on highly regulated mechano-GTPases that are oligomerization dependent and belong to the dynamin-related protein family, like *A. nidulans* DnmA. Our previous work demonstrated that H_2_O_2_ induces mitochondrial constriction, division, and remodeling of the outer membrane. Here, we show that H_2_O_2_ also induces a DnmA aggregation consistent with higher-order oligomerization and its recruitment to mitochondria. The study of this response uncovered that H_2_O_2_ induces the depolymerization and reorganization of actin as well as the critical role that cysteines 450 and 776 play in DnmA function. Our results provide new insights into the mechanisms of reactive oxygen species cell signaling and how they can regulate the dynamics of the actin cytoskeleton and the division of mitochondria and peroxisomes.

## INTRODUCTION

Our research aims to understand the signaling roles of reactive oxygen species (ROS), such as H_2_O_2_, in cell physiology and differentiation. Mitochondria (1) and NADPH oxidase enzymes (2) are major sources of ROS in eukaryotic cells and play critical roles in general cell physiology and cell differentiation in fungi (3–7). Mitochondria are dynamic organelles that change their morphology during the cell cycle and actively divide during mitosis to secure proper segregation to the daughter cells (8). A lack of mitochondrial division results in embryonic lethality in mice (9) and severe growth and developmental defects in *Aspergillus nidulans* (5), while imbalances in mitochondrial fusion and fission are related to neurodegenerative diseases, like Alzheimer’s and Parkinson’s disorders (10).

The fungus *A. nidulans*, amenable to genetic analysis, forms polarized cells that grow at their tips, called hyphae, which can differentiate asexual or sexual structures. This and the fact that growing hyphae contain relatively large mitochondria that can be easily observed using microscopy make this organism an excellent model to approach mitochondrial cell biology questions (5, 11). Using this organism, we recently reported that H_2_O_2_ induces dramatic changes in mitochondrial morphology (5, 12), including mitochondrial constriction and division and outer membrane remodeling, and that constriction and division require mitochondrial depolarization, calcium, and the endoplasmic reticulum (ER)-mitochondria encounter structure complex (11). We also identified the dynamin-like protein DnmA and its putative receptor FisA as essential for mitochondrial division and partially required for peroxisome division. Moreover, the lack of these proteins produces increased mitochondrial ROS levels and notable defects in asexual and sexual differentiation (5).

DnmA is the homolog of *Saccharomyces cerevisiae* Dnm1 (13) and animal cells Drp1 (14), which are members of the superfamily of dynamin-related proteins. This superfamily of oligomerization-dependent mechano-GTPases includes enzymes involved in multiple cellular processes such as endocytosis, mitochondrial division and fusion, and other membrane fusion and fission events (15, 16). FisA is the Fis1 homolog in *S. cerevisiae* and animal cells. In *S. cerevisiae*, Dnm1 requires the partially redundant adaptor proteins Mdv1 and Caf4 to bind to its mitochondrial receptor Fis1, while *A. nidulans* contains a single functional Mdv1/Caf4 homolog (17). Animal cells lack Mdv1/Caf4 homologs, and Drp1 recruitment appears to be independent of Fis1 in animal, plant, and apicomplexan parasite cells (18, 19). However, recent evidence showing that Fis1 contributes to Drp1 recruitment under certain conditions suggests that this role might be cell-type specific (20). Animal cells use different proteins to recruit Drp1 to the mitochondria, known as MFF, MiD49 (also known as MIEF2), and MiD51 (also known as MIEF1). Either MiD49 or MiD51 can independently recruit Drp1, and their loss affects mitochondrial division (18). Consistent with this, the expression of human Fis1 and Drp1 in a *S. cerevisiae* mutant lacking all fission proteins did not rescue mitochondrial division, while yeast Fis1 was dispensable when the Mdv1 adaptor was tethered to the mitochondrial membrane. These results highlight important similarities and differences in the basic mitochondrial fission machinery between fungi and animals (21).

**Fig 1:**
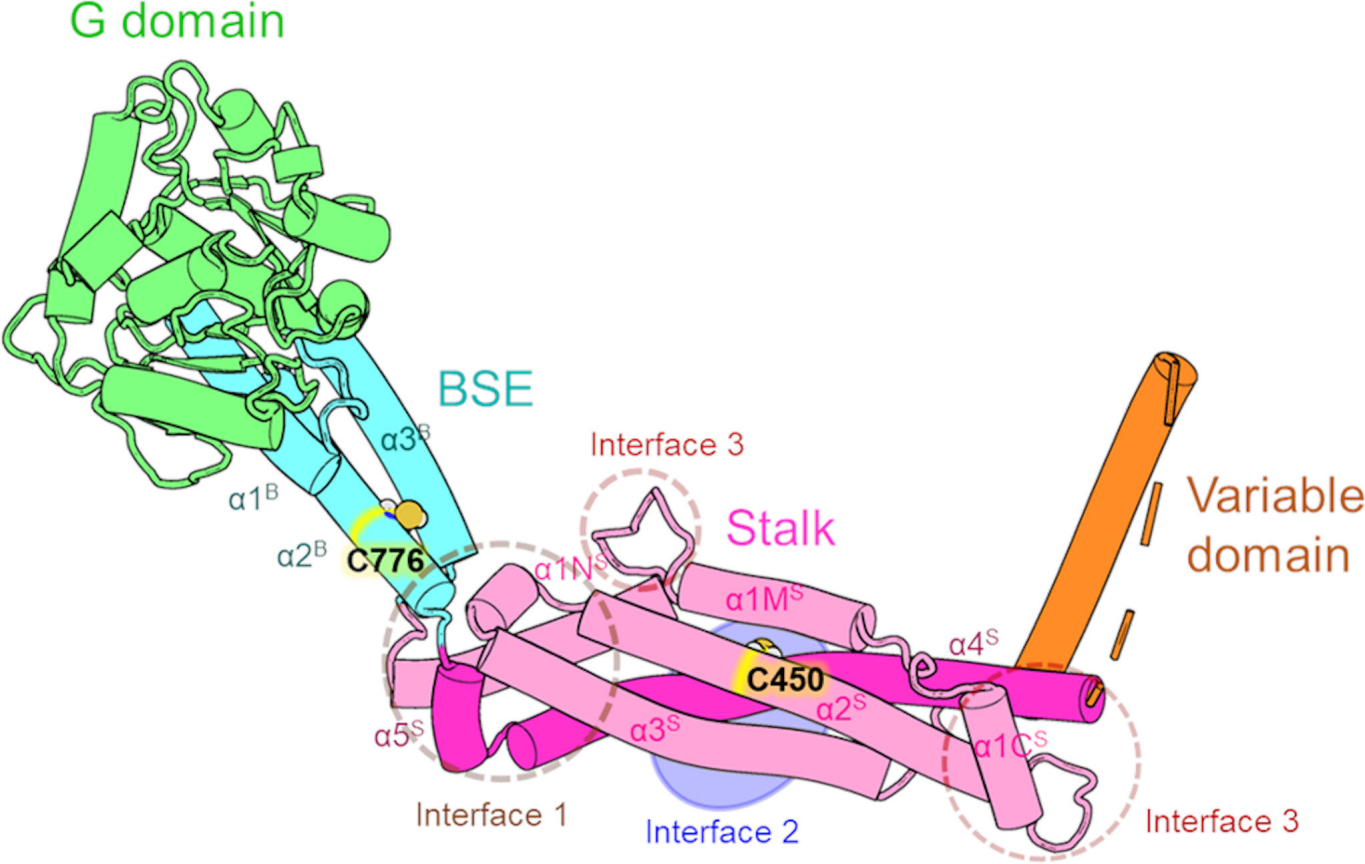
Representation of the DnmA monomer. Protein domains are shown in color, and interfaces are indicated within dashing circles. Helices are named with superscripts according to their domain. Based on DnmA AlphaFold model available at Uniprot (https://www.uniprot.org/uniprotkb/Q5AS56/entry).

All known members of the Dnm1/Drp1 family fold in a similar way to form four domains (Fig. 1), which are the GTPase (G) domain, the bundle signaling element (BSE), the stalk, and a variable domain (head, neck, trunk, and foot of the molecule, respectively) (18). The stalk is the main component involved in self-assembly. BSE is critical for intramolecular signaling and probably GTP hydrolysis (22). In Drp1, the variable domain forms an unstructured region that binds lipids such as cardiolipin (23), while the G domain and the stalk interact with the adaptor proteins (24). The stalk includes three interfaces that are critical for self-assembly. Monomers bind to each other through interface 2 to form dimers; dimers bind through interface 1 to form tetramers, while interface 3 is involved in oligomerization on the mitochondrial membrane at discrete foci, some of which develop into mitochondrial scission sites (24, 25).

In mammalian cells, Drp1 can exist in the cytosol as a mixture of different forms including dimers, tetramers, and higher-order oligomers, with receptor-mediated recruitment favoring higher-order oligomers. Once recruited to the mitochondrial membrane, Drp1 assembles into polymers that encircle the mitochondria to induce membrane scission. Drp1 structural studies have shown that the Drp1 surfaces that engage MiD49 or MiD51 receptors become available upon GTP binding and that GTP hydrolysis induces the transition of Drp1/MiD49 filaments to rings (24).

Human Drp1 is subject to multiple posttranslational modifications (26), including phosphorylation, sumoylation, and acetylation. Although Drp1 cysteine modification has not been systematically explored, cysteines 367 and 644 have been found S-nitrosylated, and it has been reported that nitric oxide increases Drp1 GTPase activity by S-nitrosylation of C644 (27, 28). Notably, Drp1 modifications are concentrated in the variable domain (sumoylation of K557, K560, K569, and K571; *O-Glc*NAcylation of T585 and T586; and phosphorylation of S616) and the GTPase effector domain (GED) (phosphorylation of S637, S-nitrosylation of C644, and acetylation of K642) (29). Likewise, many disease-causing Drp1 mutations in humans are located in the middle domain and, therefore, affect the stalk region (30, 31). Among Drp1 posttranslational modifications, it has been shown that the phosphorylation of S616 and S637 activates and inhibits mitochondrial division, respectively (32–34). Interestingly, we have detected that DnmA is phosphorylated at a serine residue (S647) equivalent to Drp1 S616 (35), suggesting that this modification might regulate DnmA function in *A. nidulans*.

In this study, we examine the impact of H_2_O_2_ on the cellular distribution of DnmA and the roles of conserved cysteines in DnmA function during *A. nidulans* growth and development as well as in mitochondrial and peroxisomal division in response to H_2_O_2_.

## RESULTS

### DnmA oligomerization and mitochondrial recruitment are affected by the presence of H_2_O_2_ and its receptor FisA

H_2_O_2_ regulates mitochondrial dynamics in *A. nidulans* (5, 12) by inducing gradual depolarization and calcium-mediated mitochondrial constrictions and division (11). To determine if H_2_O_2_ also affected DnmA subcellular distribution, we generated strain TVG5 (Fig. S1A) in which DnmA was tagged with GFP at its C-terminus and expressed from the endogenous *dnmA* promoter. In this strain, the *dnmA::gfp* gene fusion was fully sequenced to confirm the absence of any mutation. TVG5 growth, conidiation (Fig. S1B), and H_2_O_2_-induced mitochondrial division (Fig. S1C) phenotypes were indistinguishable from those observed in a wild-type (WT) strain, indicating that GFP tagging did not notably affect DnmA function.

**Fig 2:**
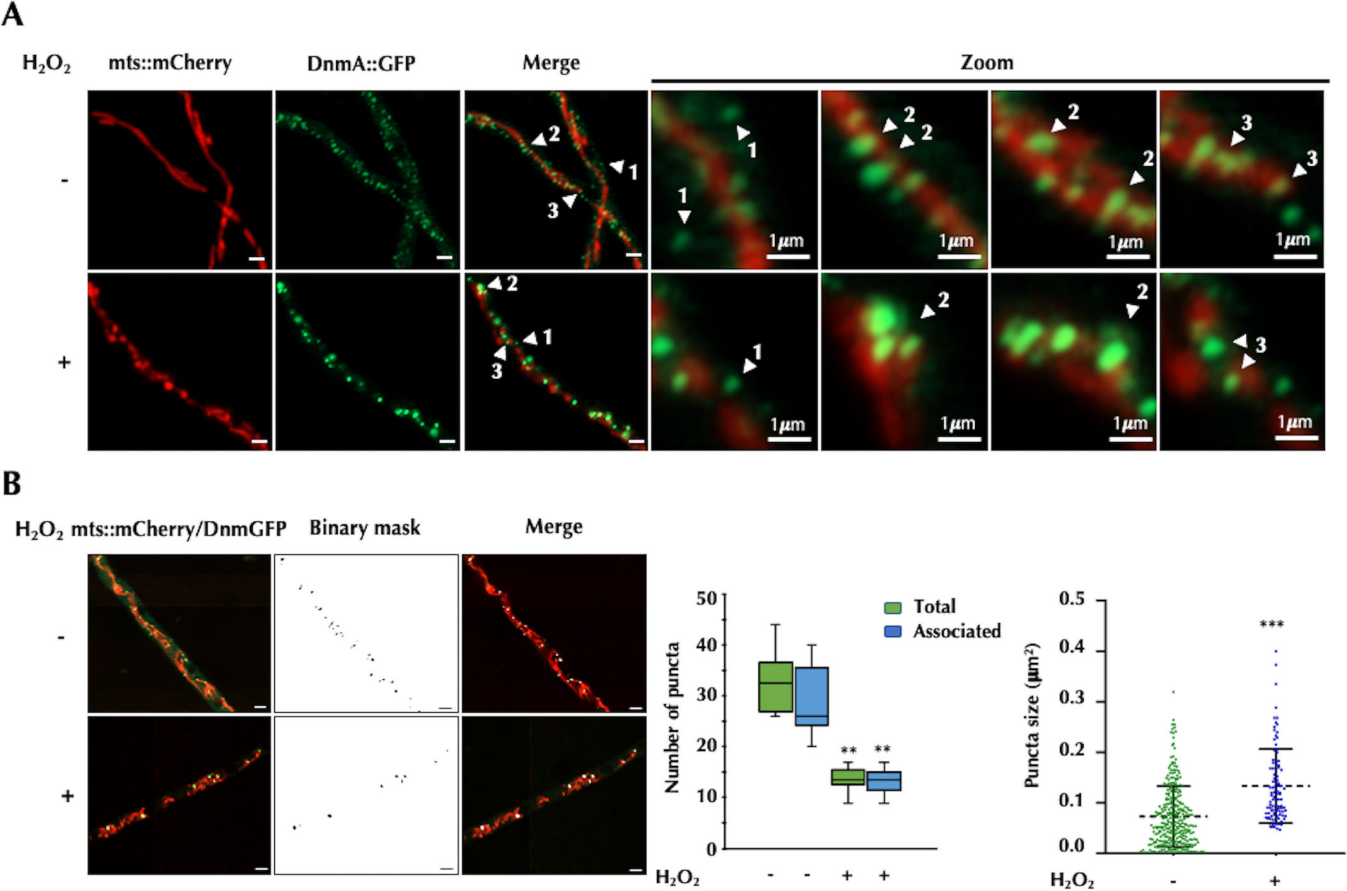
DnmA forms different-sized oligomers that are either localized in the cytoplasm or associated to dividing and non-dividing mitochondria. (**A**) Mycelia from strain CVG14 (*dnmA::gfp*) grown for 22 h were treated or not with 5 mM H_2_O_2_ for 20 min, as reported, and hyphae were observed using Airyscan microscopy. The mitochondrial matrix was labeled with mts::mCherry, and DnmA was labeled with GFP. Arrowheads point to the following: 1, cytoplasmic DnmA; 2, DnmA associated to non-dividing mitochondria; 3, DnmA associated to dividing mitochondria. The scale bar in the left panels represents 2 µm. (**B**) Images from DnmA::GFP were thresholded using the Moments algorithm (FIJI, ImageJ) and then converted into binary mask images. The tool Analyze Particles was utilized to determine the number and size of DnmA puncta in 10 hyphal tips along 30 µm. Data were analyzed by one-way analysis of variance (ANOVA), followed by Dunnett’s test (^∗^*P* < 0.05). Asterisks indicate significant differences with respect to the control condition (minus H_2_O_2_). The scale bar represents 2 µm.

Strain TVG5 was crossed to strain TRV1, which contains mCherry matrix-labeled mitochondria (5), to obtain strain CVG14 containing both labeled mitochondria and GFP-tagged DnmA. In CVG14 strain growing hyphae, DnmA::GFP was mainly observed as discrete GFP punctate structures of regular size, with some showing smaller or bigger sizes, and a faint background was observed in some cases. Most of the discrete puncta were associated to the mitochondrial network, while some smaller sized structures were found in the cytoplasm (Fig. 2A). Both cytoplasmic and mitochondria-recruited DnmA puncta were highly dynamic with some cytoplasmic puncta showing anterograde and retrograde movements (Movie S1), consistent with the actin-based transport of Drp1 reported recently (36). When mitochondrial division was induced with H_2_O_2_, the total number of DnmA::GFP puncta decreased. However, under these conditions, larger and brighter DnmA puncta were observed, which are generally associated to dividing mitochondria (Fig. 2A). To confirm and quantify this, we observed multiple hyphae treated or not with H_2_O_2_ and used a binary mask image procedure to count the number of DnmA::GFP puncta detected under these two conditions. Results in Fig. 2B confirm that a higher number of DnmA puncta were detected in the absence of H_2_O_2_, with some not associated to mitochondria, while a lower number of DnmA puncta were detected in H_2_O_2_, with most of them associated to mitochondria. They also show that DnmA::GFP puncta median size increases in the presence of H_2_O_2_.

As previously mentioned, the oligomerization process is required for enabling the GTPase and constriction activities of the dynamin-related proteins. More importantly, independent studies using Drp1-GFP *in vivo* imaging in animal cells have demonstrated that different-sized GFP puncta correspond to different Drp1 oligomeric forms (37–40). Consequently, our findings support a model in which H_2_O_2_ promotes the formation of higher-order DnmA oligomers.

**Fig 3:**
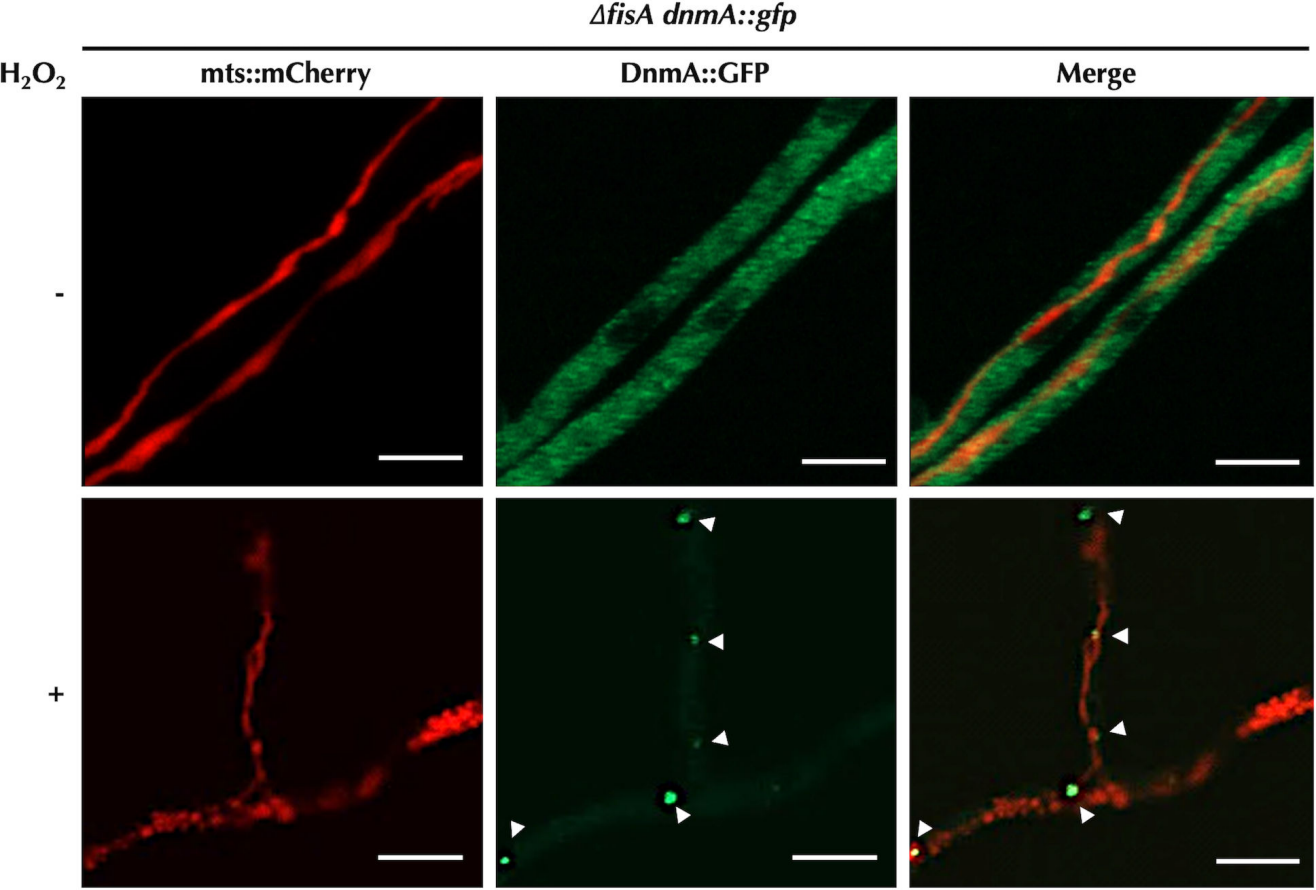
In the absence of its receptor FisA, DnmA is dispersed throughout the cytoplasm, where H_2_O_2_ induces the formation of large DnmA assemblies associated to mitochondria. Mycelia from strain CVG24 (Δ*fisA dnmA::gfp*) grown for 22 h were treated or not with 5 mM H_2_O_2_ for 20 min. H_2_O_2_ was removed, and hyphae were observed using Airyscan microscopy. The mitochondrial matrix was labeled with mts::mCherry, and DnmA, with GFP. DnmA::GFP assemblies are indicated by arrowheads. Scale bar represents 5 µm.

The general distribution of DnmA changed drastically when protein FisA was absent. As shown in Fig. 3, in FisA-lacking strain CVG24, DnmA was not associated to mitochondria, and instead, it was evenly distributed throughout the cytoplasm forming a granular pattern of much smaller puncta, along with some slightly larger ones. These small and larger puncta might correspond to DnmA dimeric and tetrameric structures, respectively. These results show that FisA is required for the mitochondrial recruitment of DnmA. Additionally, they indicate that this recruitment results in the formation of larger DnmA structures, which very likely correspond to higher-order DnmA oligomers, as it has been reported for Dnm1 in *S. cerevisiae* (21) and for Drp1 in animal cells (40). Furthermore, these findings indicate that DnmA mitochondrial recruitment by FisA does not result *per se* in mitochondrial division. In the absence of FisA, mitochondrial division cannot proceed, and as reported before (11), H_2_O_2_ induced the formation of numerous transient mitochondrial constrictions (Fig. 3; Movie S2). Notably, under these conditions, DnmA::GFP cytoplasmic signal became fainter, and some discrete and much larger DnmA puncta were formed (Fig. 3). Although these larger DnmA aggregates were highly dynamic, they moved in close proximity to the mitochondria (Movie S2; Fig. S2). These results demonstrate that even in the absence of FisA, H_2_O_2_ can induce the formation of large DnmA aggregates that likely correspond to higher-order oligomeric forms, which interact with the mitochondria through a FisA-independent mechanism.

In summary, our results are consistent with those obtained in mammalian cells, where DnmA homolog Drp1 is found in the cytosol as a mixture of forms including tetramers and higher-order oligomers, while Drp1 receptor-mediated recruitment favors the formation of higher-order oligomers (37–40). More importantly, our results show that H_2_O_2_ affects the subcellular distribution and organization of DnmA.

### Actin destabilization induced by H_2_O_2_ or latrunculin triggers the formation of DnmA large assemblies

Drp1 binds to other cellular structures besides mitochondria, such as microtubules (41) and actin, and there is evidence indicating that actin can function as a dynamic reservoir for Drp1 (42). Having shown that H_2_O_2_ affects DnmA subcellular distribution and organization, we explored the possibility that H_2_O_2_ could indirectly affect DnmA oligomerization and mitochondrial recruitment by altering the actin cytoskeleton and, therefore, the availability of DnmA.

**Fig 4:**
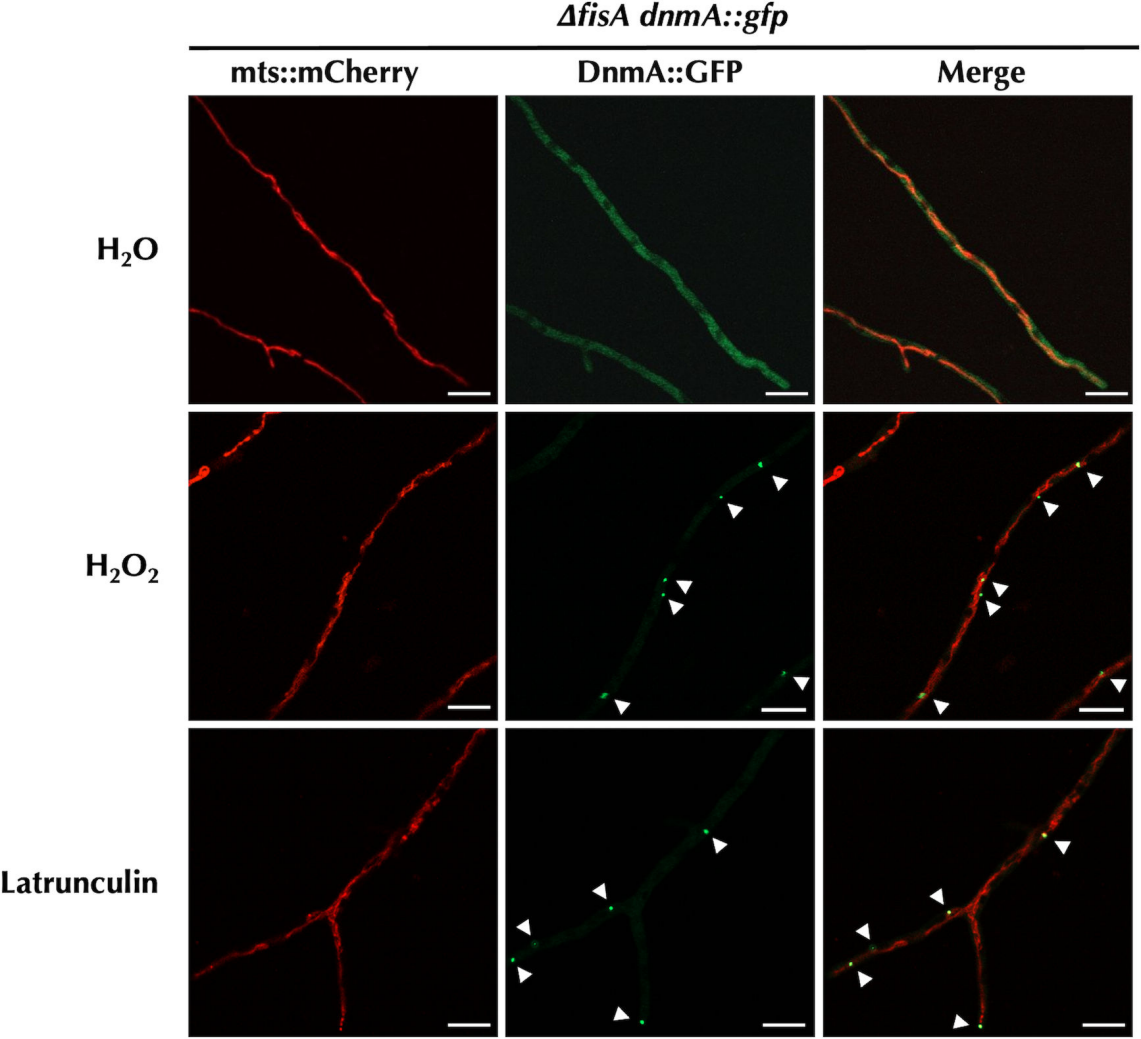
Latrunculin and H_2_O_2_ induce the formation of large DnmA assemblies in the absence of FisA. Mycelia from strain CVG24 (Δ*fisA dnmA::gfp*) grown for 22 h were treated with 22 µM latrunculin for 10 min or 5 mM H_2_O_2_ for 30 min. After this, mycelia were rinsed and observed using confocal microscopy. Arrowheads indicate DnmA::GFP large assemblies. The scale bar represents 10 µm.

**Fig 5:**
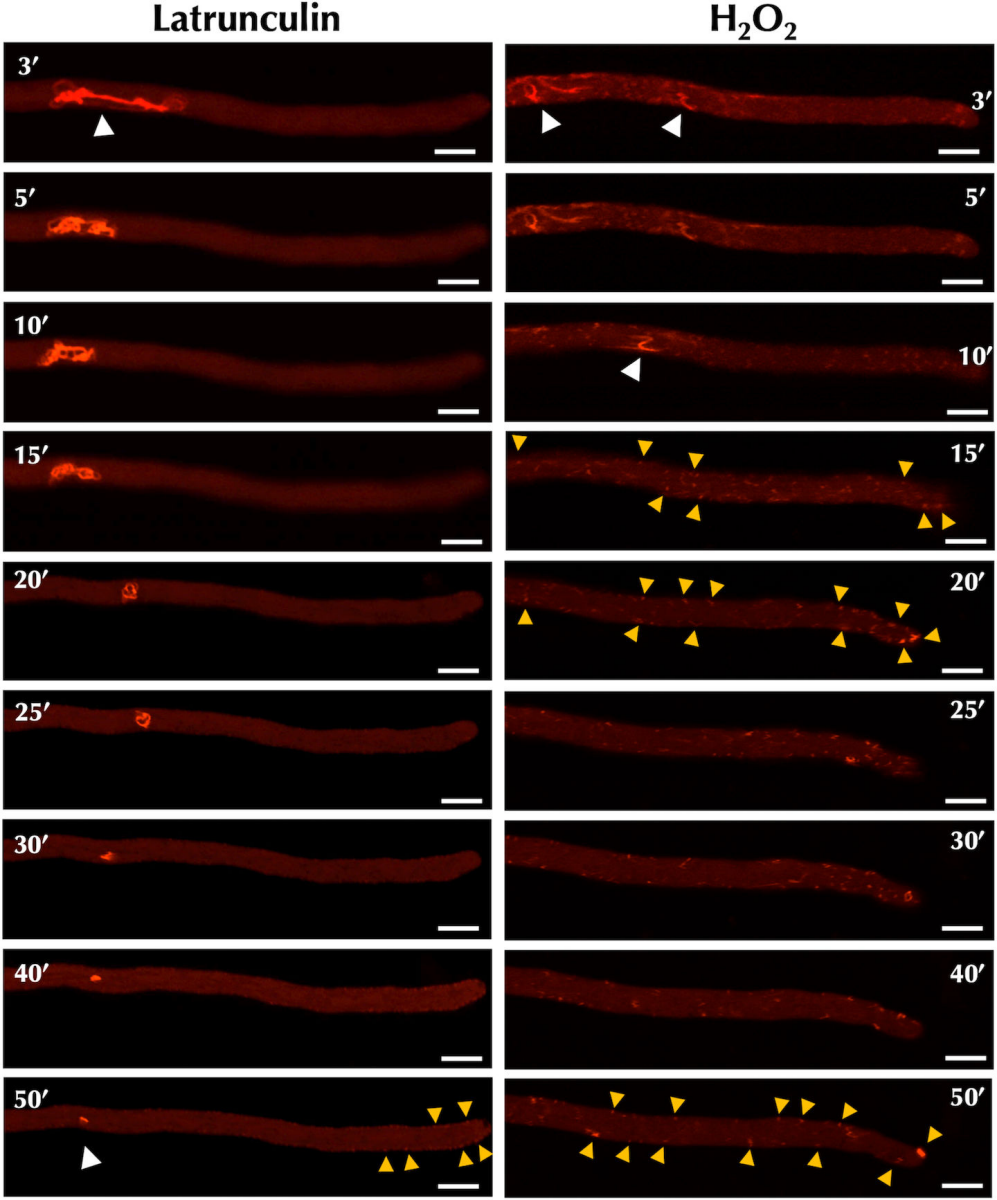
Hydrogen peroxide and latrunculin induce fast actin depolymerization of filamentous actin and the formation of actin patches. Mycelia from strain LQR3 (*LifeAct::TagRFP*) grown for 22 h were incubated with 22 µM latrunculin or 5 mM H_2_O_2_ and after 2 min, continuous *in vivo* observations were made using Airyscan microscopy. Pictures were taken at the specified time intervals. The subapical actin web is indicated by white arrowheads, while actin patches are indicated by yellow arrowheads. The scale bar represents 5 µm.

To this end, we compared DnmA::GFP organization in strain CVG24, lacking FisA, treated with H_2_O_2_ or the actin polymerization inhibitor latrunculin. Results in Fig. 4 show that, as before, H_2_O_2_ induced both mitochondrial constrictions and the formation of large DnmA aggregates. Notably, under these conditions, latrunculin also induced the formation of large DnmA aggregates without causing mitochondrial constriction. This suggested that both H_2_O_2_ and latrunculin could destabilize actin making more DnmA molecules available for oligomerization. To examine this hypothesis, we used strain LQR3, which expresses the LifeAct peptide tagged with red fluorescent protein (RFP) under the control of the *niiA* promoter (43). This allowed us to monitor actin dynamics in response to latrunculin and H_2_O_2_. As previously reported (43), we observed that in growing hyphae, actin was arranged in patches and cable networks. Cable networks were organized either as an apical actin array (Fig. S3) or as a subapical actin web located in a more distal region of the hyphal tip (Fig. 5).

Under normal conditions, without any treatment, hyphal growth was maintained, and the cable network underwent continuous reshaping, indicating actin polymerization and depolymerization events (Fig. S3). However, when growing hyphae were treated with latrunculin, the actin cable network was gradually depolymerized becoming barely detectable after 40 min of incubation, and hyphal growth was halted. Between 40 and 50 min, very small cortical actin patches were observed near the hyphal tip. Notably, H_2_O_2_ induced a fast disappearance of the actin network, which was no longer detectable after 15 min of incubation. Consistent with this, hyphal growth was also arrested. Additionally, larger actin patches compared to those detected with latrunculin were found along hyphae between 15 and 50 min of incubation (Fig. 5). These results indicate that H_2_O_2_ induces rapid depolymerization of filamentous actin and a generalized rearrangement of the actin cytoskeleton at both the hyphal tip and the subapical region, as evidenced by an increased formation of actin patches in these regions.

In summary, our results demonstrate that H_2_O_2_ induces an increased oligomerization and mitochondrial recruitment of DnmA when FisA receptor is present, and the formation of larger DnmA assemblies associated to mitochondria when FisA is absent. In both conditions, H_2_O_2_ triggers rapid actin depolymerization and other changes in actin organization. Overall, these findings support a model in which actin functions as a reservoir for DnmA, and the perturbation of the actin cytoskeleton by H_2_O_2_ or latrunculin increases the availability of DnmA for oligomerization. Furthermore, our results indicate that actin polymerization can be redox regulated in fungi.

### Cysteines 450 and 776 play distinct critical roles in DnmA function

H_2_O_2_ could potentially affect either DnmA-actin interaction, its oligomerization, or its scission activity by altering the oxidation state of specific cysteine residues. To explore this, we examined the roles of DnmA conserved cysteines in DnmA function. The protein alignment in Fig. S4 shows that DnmA homologs, including human Drp1, contain several highly conserved cysteines. Using DnmA amino acid position as reference, we identified that C295 is not present in Drp1 but is conserved in all fungal homologs included. C380 and C465 are present in Drp1 and all fungal homologs, except in *Schizosaccharomyces pombe* Dnm1. C462 is absent only in Drp1 and *Candida albicans* proteins. C776 is absent only in Drp1, and notably, C450 is conserved in all DnmA homologs. All these cysteines are located in DnmA middle domain except for C776, which is located in the GED domain (Fig. 1; Fig. S4). The regulation of human Drp1 activity by S-nitrosylation of cysteines 367 and 644 has been reported before (27, 28). However, C367 is not present in any of the fungal homologs, while C644 is present only in *C. albicans* and *Saccharomyces cerevisiae* proteins.

We designed a strategy to substitute all highly conserved cysteines in DnmA with serine, an amino acid that is similar but not sensitive to oxidation (44), especially to H_2_O_2_, to evaluate the effects of such substitutions in DnmA function. We obtained C295S, C380S, C450S, and C776S corresponding alleles. However, after multiple attempts, we could not obtain the C465S allele. As indicated before, the lack of mitochondrial division causes major defects in asexual and sexual developments (5). Results in Fig. S5 show that C295S, C380S, and C462S substitutions produced minor or no effects on growth or asexual development (Fig. S5A). Likewise, during sexual development, these mutants produced similar numbers of cleistothecia (Fig. S5B), which generated similar numbers of viable ascospores (Fig. S5C). Consistent with this, these mutations did not notably affect DnmA distribution or mitochondrial morphology during hyphal growth. Moreover, mitochondrial division was induced by H_2_O_2_ in the mutants carrying these alleles (Fig. S6). Therefore, these results indicate that these three cysteines do not play a critical role in DnmA function.

**Fig 6:**
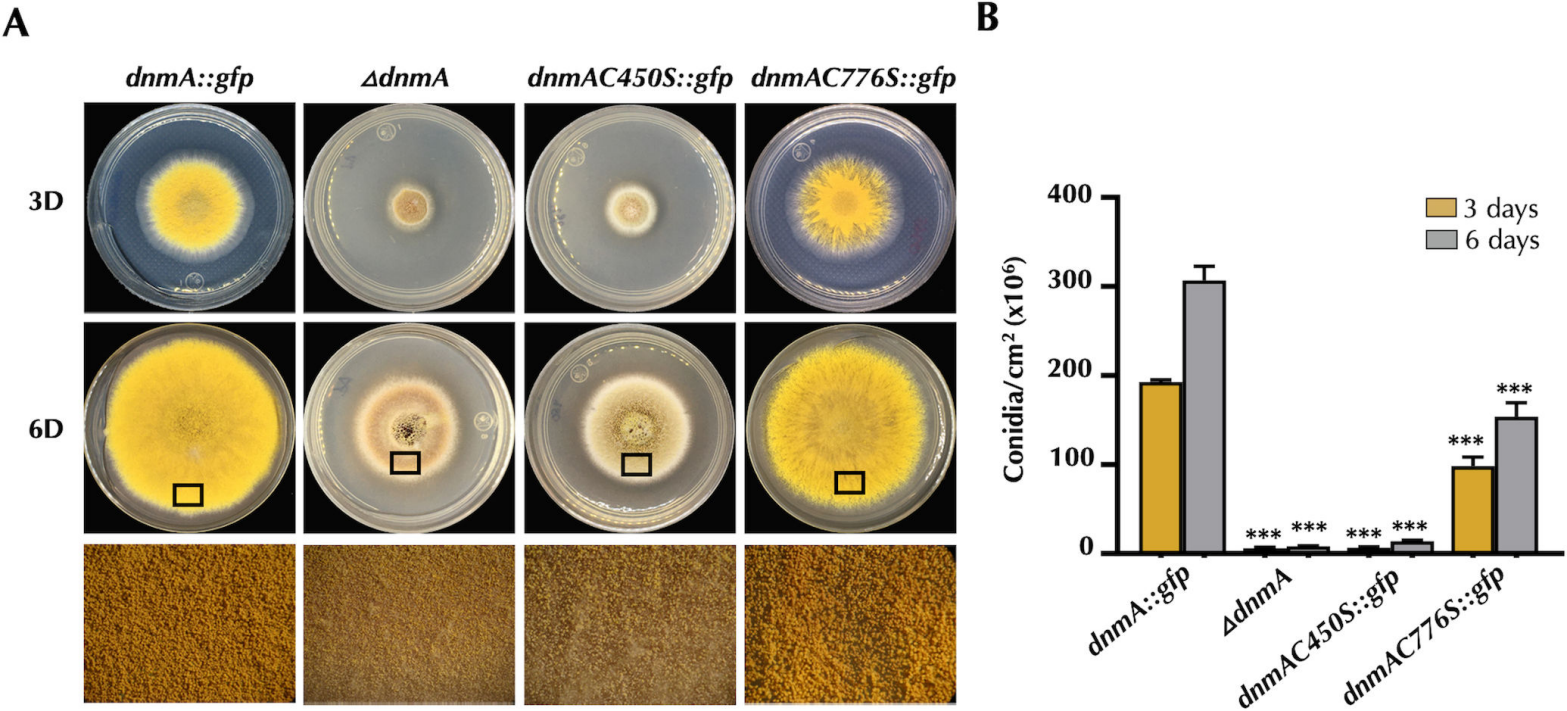
Both the absence of DnmA and the DnmA C450S substitution result in similarly severe growth and asexual development defects, while C776S replacement shows moderate defects. (**A**) Conidia (1 × 10^4^) from strains CVG14 (*dnmA::gfp*), CVG1 (*ΔdnmA*), CVG53 (*dnmAC450S::gfp*), and CVG33 (*dnmAC776S::gfp*) were used to inoculate supplemented minimal media plates and incubated at 37°C for 3 (3D) or 6 (6D) days. The lower panel shows an enlarged view of the colony edges (indicated by black squares). (**B**) The total number of conidia per colony area was quantified. The yellow and gray bars represent the number of conidia per square centimeter for 3D and 6D colonies, respectively. The standard deviation from three independent experiments is shown. Data were analyzed by one-way ANOVA, followed by Dunnett’s test (^∗^*P* < 0.05). Asterisks indicate significant differences with respect to the control strain CVG14 (*dnmA::gfp*).

**Fig 7:**
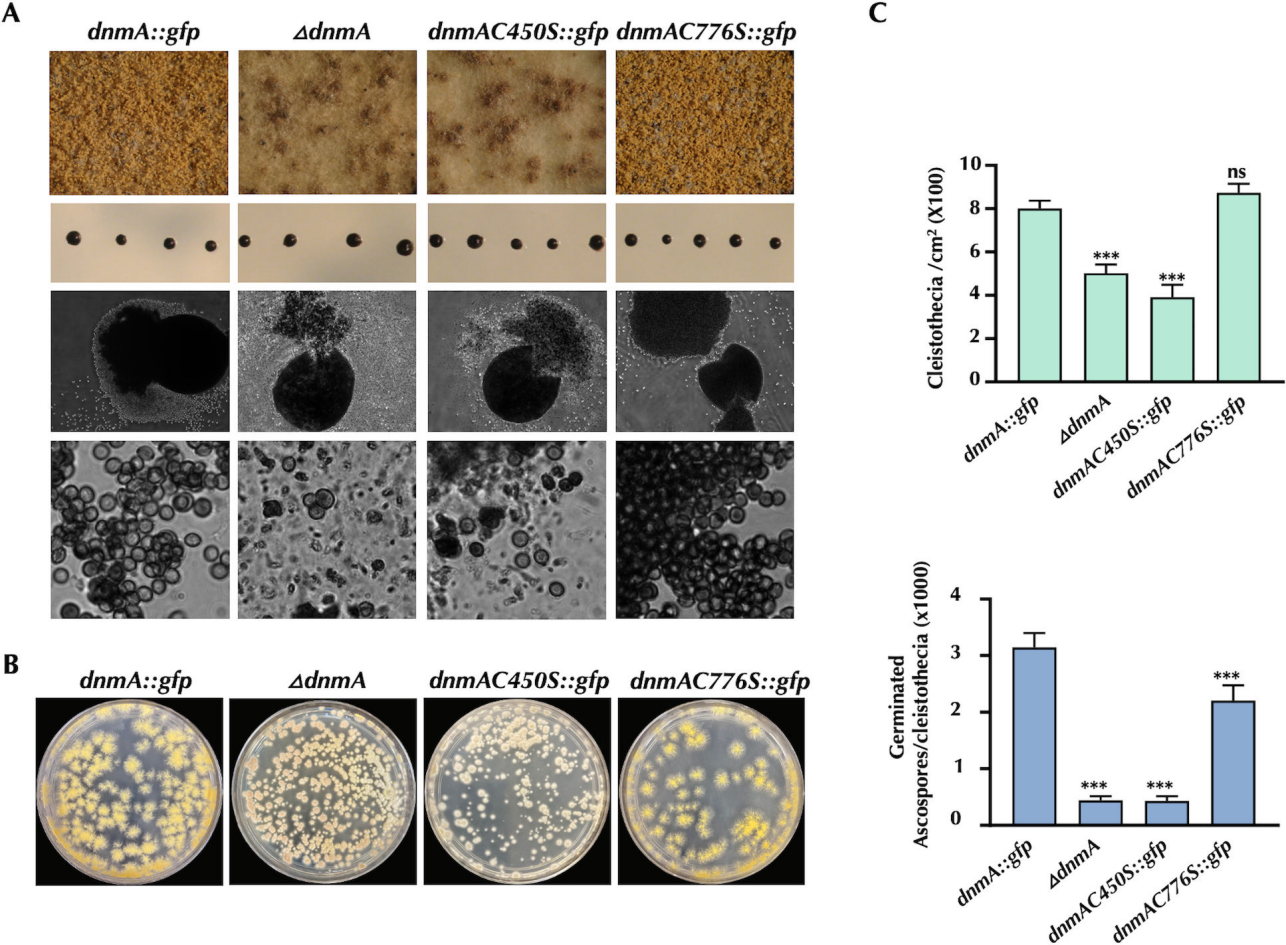
The absence of DnmA and the DnmA C450S substitution both result in similarly severe sexual development defects, whereas C776S replacement shows moderate defects. (**A**) Strains CVG14 (*dnmA::gfp*), CVG1 (*ΔdnmA*), CVG53 (*dnmAC450S::gfp*), and CVG33 (*dnmAC776S::gfp*) were induced to undergo sexual development as previously reported (45). The top panel shows a representative experiment. The number of cleistothecia per square centimeter was determined (right panel), and intact or crushed isolated cleistothecia (middle panels) from each strain grown for 8 days were observed under the microscope. The lower panel shows ascospores released from a single cleistothecium. (**B**) Single cleistothecia from each strain were crushed, and the corresponding ascospore suspensions were used to inoculate supplemented minimal media plates and quantify the number of viable ascospores per cleistothecium (right panel). The standard deviation from three independent experiments is shown. Data were analyzed by one-way ANOVA, followed by Dunnett’s test (^∗^*P* < 0.05). Asterisks indicate significant differences with respect to the control strain CVG14 (*dnmA::gfp*).

In sharp contrast, C450S and C776S replacements notably affected *A. nidulans* development and mitochondrial division. Remarkably, the mutant carrying the C450S allele showed a dramatic reduction in both radial growth and conidiation, displaying a general null phenotype very similar to the one observed in the *ΔdnmA* mutant CVG1, which completely lacks the DnmA protein (Fig. 6A and B). During sexual development, the C450S mutant produced fewer cleistothecia than the wild-type strain, which produced ascogenous sterile tissue and few viable ascospores. Again, this phenotype was virtually identical to the one displayed by the *ΔdnmA* mutant (Fig. 7A through C). In the C776S mutant, conidiophore formation was heterogeneous, particularly after 3 days of growth, and although radial growth was not drastically affected, the colonies formed irregular borders. Although not as severely as in the C450S mutant, asexual spore formation was notably reduced when compared to the *dnmA::gfp* strain (Fig. 6A and B). During sexual development, C776S mutant developed a number of normal fruiting bodies similar to those generated by the reference strain *dnmA::gfp*. However, these cleistothecia produced about 30% less viable ascospores than the *dnmA::gfp* strain (Fig. 7A through C).

**Fig 8:**
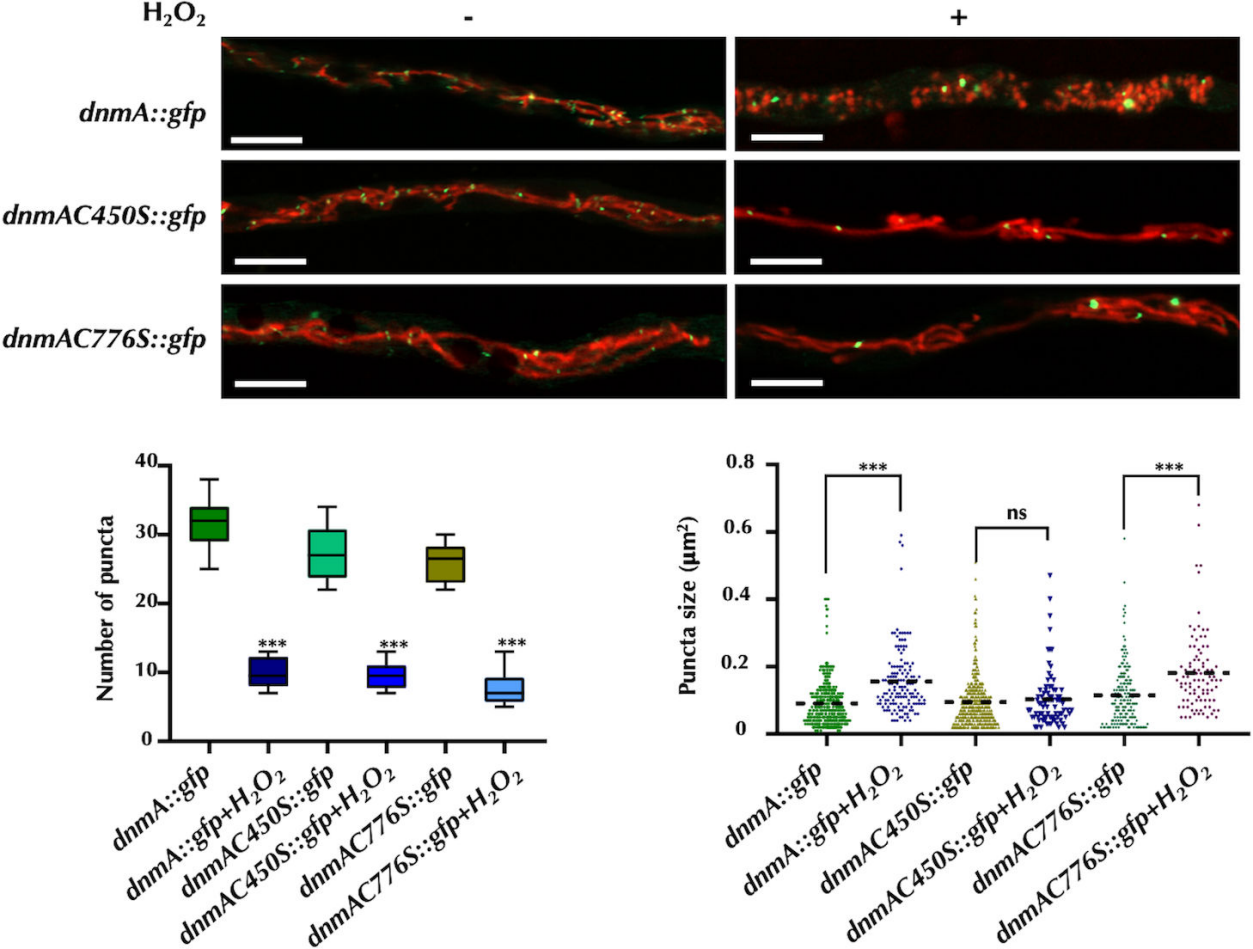
DnmA cysteines 450 and 776 are both essential to induce mitochondrial fission in response to H_2_O_2_. Mycelia from strains CVG14 (*dnmA::gfp*), CVG53 (*dnmAC450S::gfp*), and CVG33 (*dnmAC776S::gfp*) grown for 22 h were treated or not with 5 mM H_2_O_2_ for 20 min. H_2_O_2_ was removed, and mycelia were observed using Airyscan microscopy. Ten hyphal tips per condition were used to determine the number and area of DnmA::GFP puncta. The graphs present data from three independent experiments. Data were analyzed by one-way ANOVA, followed by Dunnett’s test (^∗^*P* < 0.05). Asterisks indicate significant differences with respect to the control condition (minus H_2_O_2_). The scale bar represents 5 µm.

Regarding DnmA distribution and mitochondrial division, C450S replacement caused a slight reduction in the number of DnmA::GFP puncta in growing hyphae. Nevertheless, a similar number of puncta were observed in C450S and *dnmA::gfp* strains in H_2_O_2_. However, H_2_O_2_ did not seem to induce the formation of larger DnmA puncta, and more importantly, mitochondria failed to divide under these circumstances (Fig. 8). Growing hyphae from the C776S mutant displayed a similar number of DnmA::GFP puncta as detected in the strain carrying the WT DnmA::GFP fusion. Similar to the WT strain, the presence of H_2_O_2_ reduced the number of puncta. However, the puncta in the C776S mutant were larger in size compared to those observed in the *dnmA::gfp* strain exposed to H_2_O_2_. Notably, H_2_O_2_ also failed to induce mitochondrial division in the C776S mutant (Fig. 8).

**Fig 9:**
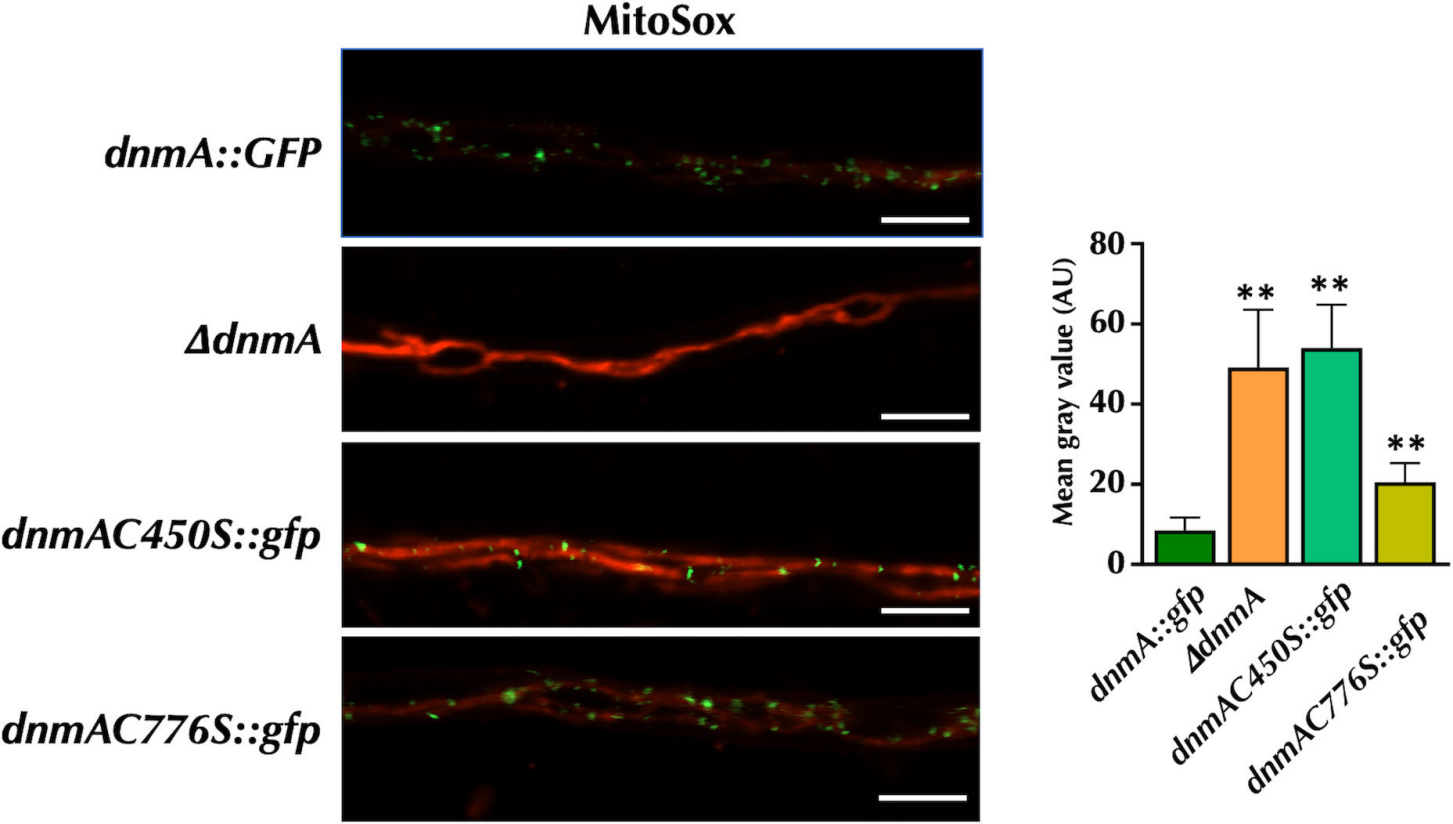
The absence of DnmA and the DnmA C450S substitution both result in similarly high mitochondrial ROS levels, while C776S replacement shows moderate ROS levels. Mycelia from strains TVG5 (*dnmA::gfp*), TVG16 (*dnmAC450S::gfp*) and TVG7 (*dnmAC776S::gfp*) were grown for 22 h and incubated with 5 µM MitoSOX for 20 min at 37°C. After removing the solution, mycelia were immediately observed using confocal microscopy. Mean gray values from 10 hyphal tips from each strain were used for fluorescence quantification. Data were analyzed by one-way ANOVA, followed by Dunnett’s test (^∗^*P* < 0.01). Asterisks indicate significant differences with respect to control strain TVG5. The scale bar represents 5 µm.

The absence of mitochondrial division leads to elevated levels of mitochondrial ROS (5). To further validate the impact of DnmA C450S and C776S substitutions on mitochondrial division, we utilized MitoSOX staining, as reported before (5), to monitor mitochondrial ROS levels in the respective mutants. Figure 9 shows that hyphae from the WT *dnmA::gfp* strain exhibited minimal MitoSOX staining under the tested conditions. In contrast, hyphae from both *ΔdnmA* and *dnmAC450S::gfp* mutants displayed similar and significantly higher staining. The hyphae from *dnmAC776S::gfp* strain showed a MitoSOX staining intensity level significantly higher than that observed in the wild type but lower than that in the *ΔdnmA* and *dnmAC450S::gfp* mutants. These results are consistent with the fact that both DnmA C450S and C776S substitutions result in major defects in mitochondrial division. However, the C776S substitution might allow for some degree of division, thereby preventing a higher accumulation of mitochondrial ROS.

**Fig 10:**
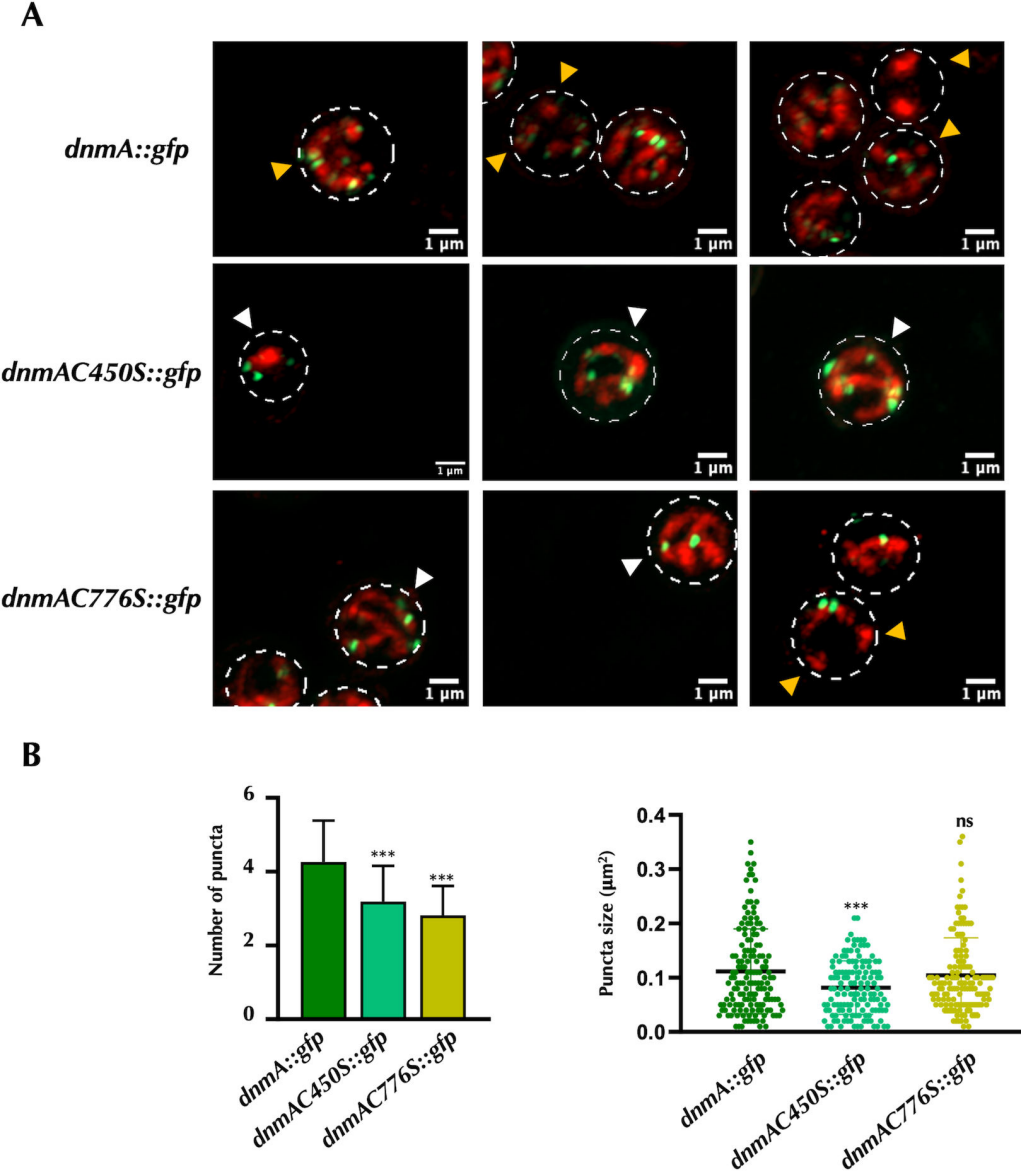
Conidia from DnmA C450S mutant contain undivided mitochondria, while some divided mitochondria are observed in conidia from the C776S mutant. Isolated conidia from strains CVG14 (*dnmA::gfp*), CVG53 (*dnmAC450S::gfp*), and CVG33 (*dnmAC776S::gfp*) were observed using Airyscan microscopy. The images correspond to maximum intensity Z projections. Single mitochondrion or single undivided mitochondrial networks are indicated by white arrowheads, while divided mitochondria are indicated by yellow arrowheads. The number and area of DnmA puncta were determined in 50 conidia per strain. Data were analyzed by one-way ANOVA, followed by Dunnett’s test (*P < 0.05). Asterisks indicate significant differences with respect to control strain CVG14. Scale bar represents 1 µm.

To further investigate this point, we examined mitochondrial division in C450 and C776S mutants under asexual sporulation conditions. In *ΔdnmA* null mutants, mitochondrial division is totally impaired in response to H_2_O_2_, and by a DnmA-independent mechanism, these mutants produce a reduced number of conidia that contain a single mitochondrion or mitochondrial network (5). Results in Fig. 10 show that a strain carrying the *dnmA::gfp* alleleformed conidia that, like WT conidia (5), contained multiple individual mitochondria. Conversely, conidia from *dnmAC450S::gfp* mutant, similar to *ΔdnmA* mutants, contained either a single small mitochondrion or a larger single undivided mitochondrial network. The *dnmAC776S::gfp* mutant also displayed undivided mitochondria in conidia, but divided mitochondria were detected in some cases. Compared to the *dnmA::gfp* strain, the number of DnmA puncta per conidia slightly decreased in *dnmAC450S::gfp* and *dnmAC776S::gfp* strains, while the size of DnmA puncta was slightly smaller in *dnmAC450S::gfp* conidia. These results confirm that C450S DnmA substitution completely abolishes DnmA function both in response to H_2_O_2_ and during growth and development. In contrast, C776S replacement produces a DnmA protein unresponsive to H_2_O_2_ but partially responsive to other DnmA regulatory inputs during growth and asexual development. This would explain that the C776S mutant shows less drastic growth and developmental defects and moderate levels of mitochondrial ROS.

### C450S and C776S substitutions have opposite effects on DnmA oligomerization

**Fig 11:**
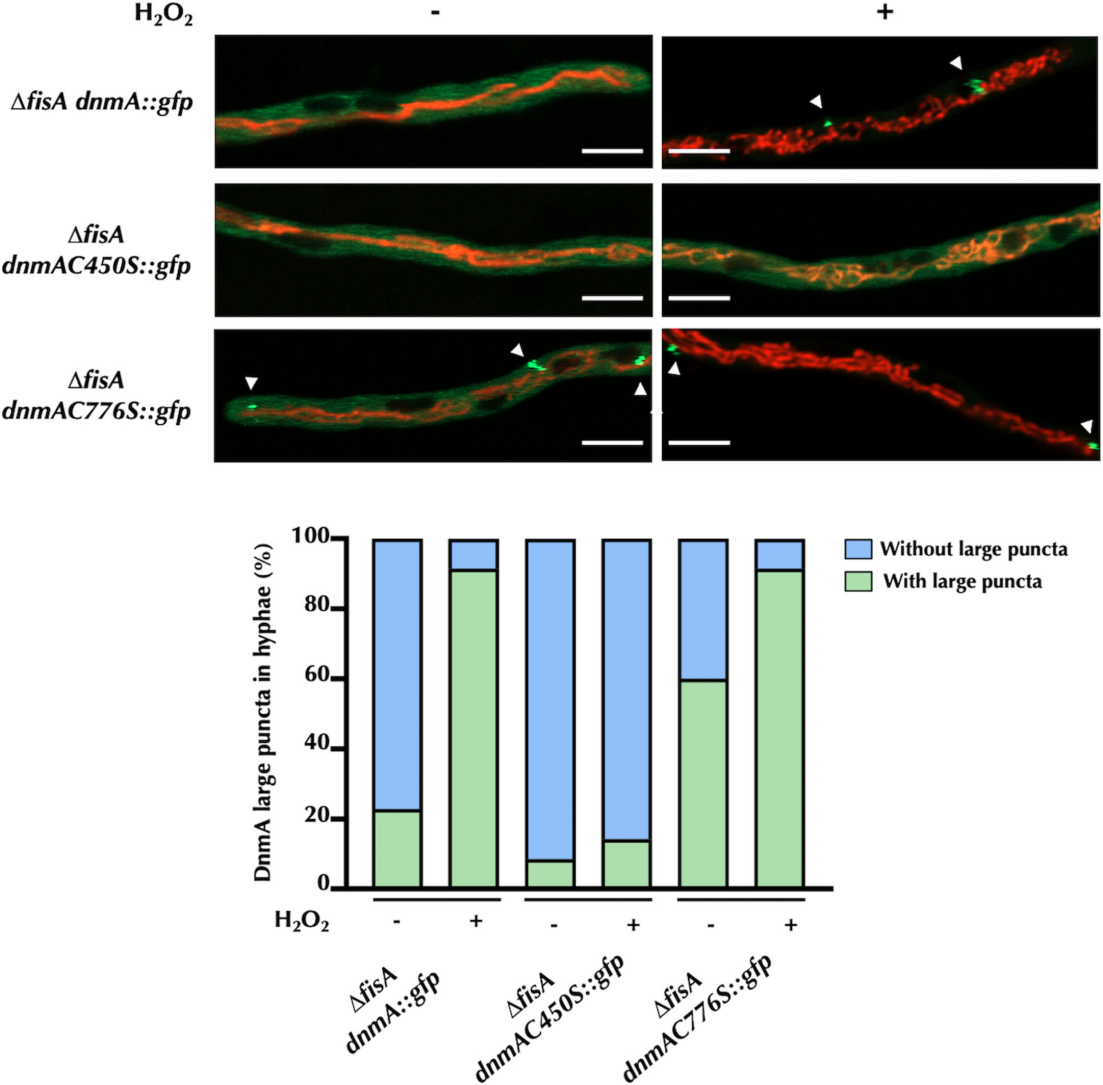
In absence of FisA, DnmA C450S and C776S substitutions result in delayed and premature DnmA oligomerization, respectively. Mycelia from strains CVG24 (Δ*fisA dnmA::gfp*), CVG58 (Δ*fisA dnmAC450S::gfp*), and CVG43 (Δ*fisA dnmAC776S::gfp*) were grown for 22 h and treated with or without 5 mM H_2_O_2_ for 20 min. After removing H_2_O_2_, hyphae were observed using Airyscan microscopy. The presence or absence of DnmA puncta was counted in 35 hyphal tips from each condition. DnmA oligomers are indicated by white arrowheads. The scale bar represents 5 µm.

We also examined the effect of H_2_O_2_ on DnmA C450S and DnmA C776S oligomerization, in the absence of FisA. Results in Fig. 11 show that without H_2_O_2_, only about 20% of *ΔfisA dnmA::gfp* hyphae contained at least one large DnmA aggregate, while this number increased to 90% after a 20-min H_2_O_2_ treatment. In sharp contrast, H_2_O_2_ failed to induce the formation of large DnmA aggregates in the *ΔfisA dnmAC450S::gfp* mutant, which appeared only after prolonged treatment (Fig. S7). Notably, DnmA::GFP C776S was already organized in large assemblies in about 60% of the hyphae before the H_2_O_2_ treatment, and this number increased to about 90% after H_2_O_2_ was added.

These results indicate that C450S and C776S substitutions have opposite effects on DnmA oligomerization and that while C450S delays oligomerization, C776 promotes it. More importantly, they indicate that these defects on DnmA oligomerization have profound effects on its function on mitochondrial division.

### C450S and C776S mutants are similarly affected in peroxisomal division

**Fig 12:**
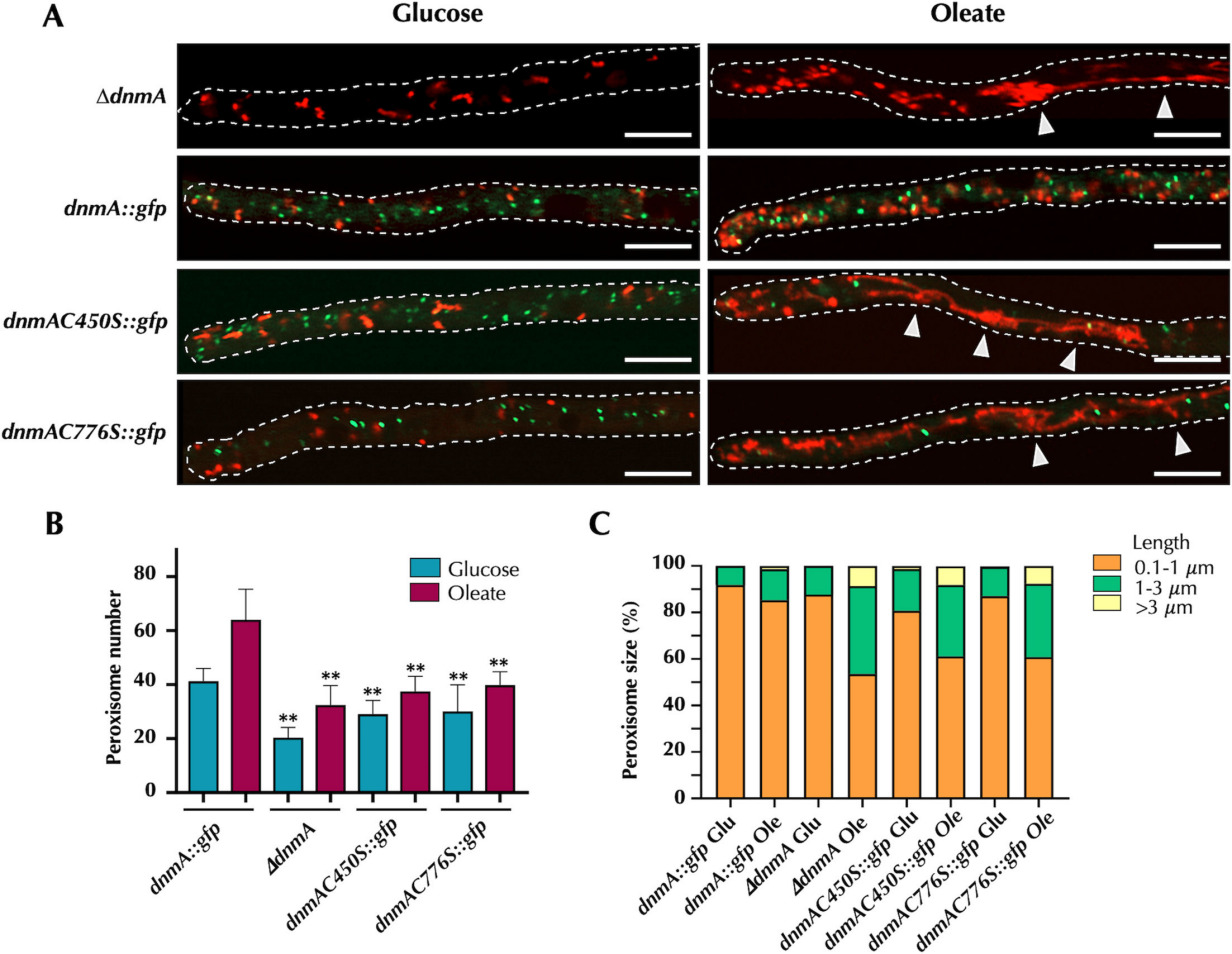
DnmA cysteines 450 and 776 are required for peroxisomal division. (**A**) Strains CVG30 (*ΔdnmA* mcherry::FLAG-PTS1), CVG59 (*dnmA::gfp* mcherry::FLAG-PTS1), CVG55 (*dnmAC450S::gfp* mcherry::FLAG-PTS1), and CVG56 (*dnmAC776S::gfp* mcherry::FLAG-PTS1) were grown for 22 h using glucose or oleate as sole carbon sources. After this period, hyphae were observed using Airyscan microscopy. The total number of peroxisomes (B) and the length of peroxisomes (C) were determined in 10 hyphal tips per condition. Data in panel **B** were analyzed by one-way ANOVA, followed by Dunnett’s test (*P < 0.05). Asterisks indicate significant differences with respect to control strain CVG59. Larger and filamentous peroxisomes are indicated by white arrowheads. The scale bar represents 5 µm.

As reported before, DnmA also plays a significant role in peroxisome division when *A. nidulans* utilizes oleate as the sole carbon source (5). To assess the impact of DnmA C450S and C776 substitutions on peroxisomal division, we generated strains CVG55 and CVG56. These strains carry the *dnmAC450S::gfp* and *dnmAC776S::gfp* alleles, respectively, along with mCherry-labeled peroxisomes. The results depicted in Fig. 12A and B indicate that compared to the wild-type *dnmA::gfp* strain, mutants lacking DnmA (*ΔdnmA*) or containing *dnmAC450S::gfp* or *dnmAC776S::gfp* alleles exhibited lower peroxisome numbers under both glucose and oleate conditions. The number of DnmA puncta also was decreased in *dnmAC450S::gfp* and *dnmAC776S::gfp* mutants when grown on oleate. More importantly, all three *dnmA* mutants displayed notable alterations in peroxisome morphology, characterized by the formation of increased size or tubular peroxisomes, with some showing constrictions and convoluted shapes (Fig. 12A and C). These findings indicate that both DnmA C450S and C776S substitutions resulted in defects in peroxisomal division similar to those caused by a complete lack of DnmA.

Several human disease-causing mutations in Drp1 exhibit heterozygous dominant-negative effects (46). To determine if C450S and C776S mutations behaved as dominant negatives, we conducted diploid complementation tests. As reference, we used a diploid strain with one wild-type *dnmA* allele and the fully functional *dnmA::gfp* allele as well as a diploid strain carrying two copies of the *dnmAC450S::gfp* mutant allele. The results in Fig. S8A demonstrate a significant reduction in radial growth only in the *dnmAC450S::gfp/dnmAC450S::gfp* diploid, with conidiation levels reaching about 35% of the wild-type *dnmA/dnmA::gfp* diploid. More importantly, H_2_O_2_ failed to induce mitochondrial division in this strain, confirming the loss of function associated with the *dnmAC450S::gfp* allele (Fig. S8B). In contrast, heterozygous diploids carrying *dnmAC450S::gfp* together with either *dnmA::gfp* or the wild-type *dnmA* allele as well as the diploid carrying *dnmAC776S::gfp and dnmA* displayed radial growth and conidiation phenotypes similar to those observed in the wild-type diploid *dnmA/dnmA::gfp*. Consistent with this, H_2_O_2_ induced mitochondrial division in all these other diploids (Fig. S8B). These findings indicate that the *dnmAC450S* and *dnmAC776S* mutations do not act as dominant negatives and suggest the possibility of DnmA450S and DnmAC776S monomers associate *in vivo* with wild-type DnmA monomers to form functional hybrid oligomers.

### C450S and C776S substitutions and C450 oxidation cause localized and global effects on DnmA structure

Before evaluating the effects of cysteine substitutions in DnmA structure, we first estimated the deprotonation probability of C450 or C776 using the PropKa server. Table S3 shows the predicted pKa values for all conserved DnmA cysteine residues. The result shows that these cysteines are rarely deprotonated in aqueous solution, while pCysMod cysteine modification analysis predicts that C450 is a candidate for S-sulfinylation with a 0.56% false-positive rate (FPR), while C776 S-sulfenylation and S-sulfinylation have a high percent FPR (Table S4). Since this indicates that C450 has a high probability of being oxidized, we performed 500 ns of traditional molecular dynamics (MD) simulations of the WT, C450S, C450 sulfinylated (C450-SO_2_^−^), and C776S systems. The root mean square deviation (RMSD) and root mean square fluctuation (RMSF) of the corresponding DnmA stalk domains are plotted in Fig. S9. Based on the RMSD plot, we discarded the first half of the simulations, so the analysis presented below was performed on the second 250 ns. The RMSF reveals the rigidity of the stalk showing the order C450S > WT > C450-SO_2_^−^ > C776S. This indicates that compared to WT, DnmA, C776S, and C450S mutations have an inverse effect on the rigidity of the stalk. A certain flexibility is required for the assembly process; therefore, the trend of the stalk rigidity may be related to the opposite effects we observed that the C450S and C776S mutations have on oligomerization.

Notably, the secondary structure (SS) analysis of stalk and BSE domains, where C450 and C776 are located, shows major changes in helices α1M^S^ and α2^S^ in the C450-SO_2_^–^ system, as shown in Fig. S10. The helix structure from residues Val392 to Gly394 in α1M^S^ gets lost, while residues Asn395 to Ile400 undergo an alpha to 3–10 helix transition due to C450 oxidation (Fig. S10A and B). These changes are promoted by the hydrogen bond (HB) interaction between the sulfinate group and the Gly394 backbone and Ser396 side chain of the α1M^S^ helix (Fig. S10C). The sulfinate group interacts with these residues 91.1% of the simulation time and 39.6% with both residues at the same time. In addition to the changes in α1M^S^ SS, C450-SO_2_^–^ modification also affects α2^S^ SS; specifically, the alpha-helical structure of the Leu430-Lys441 fragment of this system is stabilized.

The SS of α2^S^ shows that this helix bends in Leu443. Something similar happens with α3^S^ and α4^S^, the other long helices in the stalk. From now on, we will call these bending angles S2, S3, and S4, respectively. Interestingly, α5^G^ and α2^B^ helices are derived from a single helix that folds into two domains, and its bending angle (BG) varies depending on the explored modifications of the protein. The distribution of these four angles, shown in Fig. S11, reveals two very interesting facts. First, the oxidation of C450 changes all measured angles between the helices, with the BG of S2 and S4 increasing and S3 decreasing. The increment in the S2 can be related to the HB interaction between the sulfinate group and α1M^S^. The straightening of α3^S^ and the bending of α4^S^ stand out because these two helices form interface 2, which is the interface involved in the formation of the DnmA X-shaped dimers (15). The second interesting point is that contrary to what we expected, C450S mutation perturbs BG, where the C776 location is nearest, and C776S mutation perturbs S2, where C450 location is nearest. So, there is an inverse correlation between where these residues are and the bending angles they affect.

**Fig 13:**
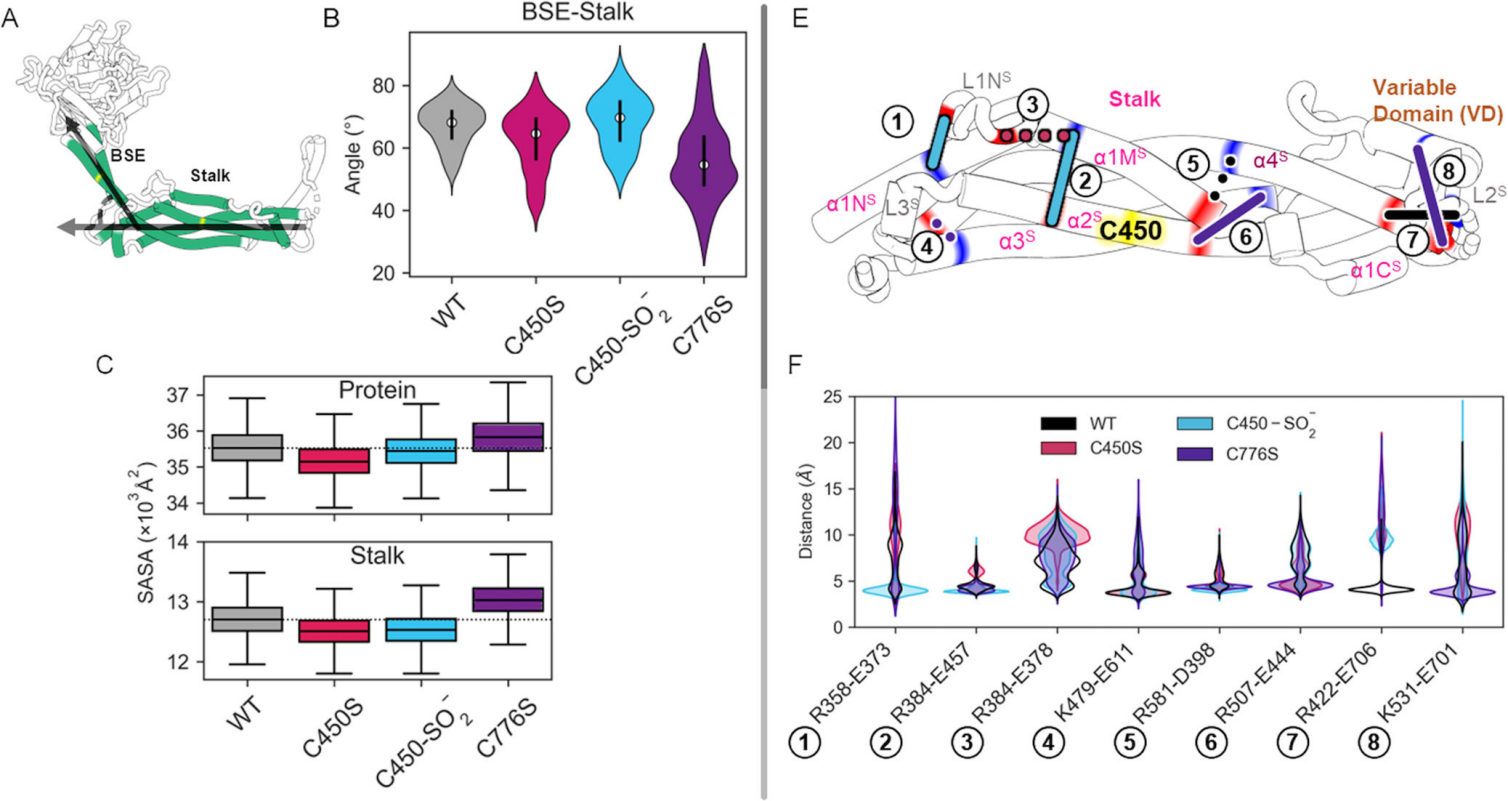
C450S, C450-SO2^−^, and C776S modifications have differential impacts on DnmA BSE-stalk angle, solvent-accessible surface area, and salt bridge interactions. (**A**) Representation of the angle between DnmA BSE and stalk domains. (**B**) Distribution of the angle between the BSE and stalk domains. (**C**) SASA distribution of the whole protein and stalk for each of the simulated systems. (**E**) Representations of key salt bridges, line colors indicate the system that is mostly impacted by each of the salt bridges, and the line style indicates a loss (dotted) or gain (solid) of interaction. (**F**) Distribution of the distances for key salt bridge interactions.

To understand how DnmA protein modifications impact its overall structure in the simulated systems, we measured an angle between the BSE and the stalk domains, as represented in Figure 13. The BSE-stalk angle decreases on C450S and C776S systems but, to a greater extent, on the C776S system. A decrease in this angle implies that the protein is expanding, since the protein in the C776S system is more expanded, its solvent-accessible surface area (SASA) values are higher, as expected. In contrast, the SASA values of the C450S system decreased, which means that the angle between the BSE and the stalk is not the only factor that modifies SASA, and the stalk itself may be contracted as suggested by the lower RMSF. Interestingly, this inverse SASA behavior may be related to the inverse oligomerization behavior that we observed in these mutants.

As shown in Fig. 13, the L2^S^-α4^S^ (Lys422-Glu706) salt bridge interaction is only present in the WT system; meanwhile, α4^S^-α1M^S^ (Arg581-Asp398) interaction is more labile in this system. The C450S system does not stabilize any salt bridge; in particular, α1M^S^-L1N^S^ (Arg384-Glu378) interaction is weakened by this mutation. This salt bridge constrains the L1NS, and its weakening could affect oligomerization, in which this loop participates. The α1N^S^-L1N^S^ (Arg358-Glu373) and α1M^S^-α2^S^ (Arg384-Glu457) interactions are favored by the S-sulfinylation of C450. α1M^S^-α2^S^ interaction complements the C450-SO_2_^−^ hydrogen bond with Gly394 and Ser396. L1N^S^ is part of interface 3 which participates in the oligomerization, and the α1N^S^-L1N^S^ interaction may constrain it. Finally, α3^S^-α2^S^ (Arg507-Glu444) and α4^S^-VD helix (Lys531-Glu701) interactions are favored by C776S mutation, and α3^S^-α4^S^ (Lys479-Glu611) interaction is slightly destabilized by it. In summary, no salt bridge is favored by the C450S mutation, while C450 sulfinylation and C776S mutation affect opposite sides of the stalk.

Taken together, these results make important predictions for the roles that DnmA C450 and C776 have in protein overall structure and oligomerization. Notably, even though C450 and C776 are located in different domains, their substitution by serine impacts both the structure of the stalk domain, critical for oligomerization, as well as the overall protein structure. Specifically, C776S induces larger changes in the angle between the stalk and the BSE domain. BSE is critical for intramolecular signaling and probably GTP hydrolysis force transfer (22). These predictions are consistent with the fact that both mutations have severe effects on DnmA function and with the fact that in the absence of FisA, C450S delays oligomerization, while C776S promotes it. Interestingly, C450 shows a high probability of oxidation, and the MD simulation of this modification predicts changes in stalk domain structure and bending angle of specific helices that would impact DnmA oligomerization (Fig. 13; Fig. S10 and 11).

## DISCUSSION

Mitochondrial division is a pivotal process in eukaryotic cell biology, and therefore, it is regulated at multiple levels to ensure proper cellular function. We have previously shown that H_2_O_2_ induces mitochondrial constriction and division in *A. nidulans*. It was also found that the Drp1 homolog DnmA and its putative receptor FisA are essential for mitochondrial division and participate in peroxisomal fission in this fungus. Additionally, a close contact between mitochondria and the ER was shown to be necessary for mitochondrial division, as previously reported (11, 47). Although the specific mechanisms targeting specific mitochondrial sites for division are not fully understood, it is known that actin polymerization at the ER-mitochondria interface and the levels of the DnmA homolog Drp1 bound to mitochondria play critical roles in this process (48, 49) and that self-assembly, oligomerization, and mitochondrial recruitment of Drp1/DnmA homologs determine their functional output. However, not all mitochondrially bound Drp1 oligomers are engaged in division, so it is important to understand how the formation of productive Drp1/DnmA oligomers is regulated. Therefore, in this study, we analyzed the impact of H_2_O_2_ on DnmA cellular distribution and evaluated the roles of four conserved cysteines in mitochondrial division.

We found that under growing conditions, some DnmA molecules show anterograde and retrograde movements in the cytoplasm, consistent with their translocation along filamentous actin, which has recently been shown for Drp1 retrograde transport (36). However, most DnmA molecules form larger structures recruited to mitochondria by the FisA receptor. The presence of H_2_O_2_ leads to the formation of fewer but larger DnmA puncta indicating that this oxidizing agent promotes DnmA oligomerization and mitochondrial recruitment. In the absence of FisA, DnmA is found dispersed throughout the cytoplasm. However, even in this condition, H_2_O_2_ induces the formation of fewer and yet larger DnmA aggregates that associate to mitochondria. Interestingly, these aggregates remain in close proximity to mitochondria, despite their high mobility and the lack of FisA. Although the oligomeric structure of DnmA within these larger assemblies is presently unknown, their formation clearly indicates that H_2_O_2_ induces alterations in DnmA that impact its self-assembly.

The DnmA anterograde and retrograde movements, along with the observation that both latrunculin and H_2_O_2_ provoked the formation of DnmA assemblies and induced the depolymerization of actin, suggest that part of the H_2_O_2_-induced oligomerization of DnmA might be attributed to the release of actin-transported DnmA, which would increase DnmA availability for mitochondrial recruitment. In our attempts to demonstrate an *in vivo* DnmA-actin interaction, we made multiple unsuccessful efforts to generate strains with labeled DnmA and actin. However, it is important to acknowledge the significant limitations associated with such approach. First, while there is conclusive evidence of the Drp1-actin interaction in animal cells, validating this interaction *in vivo* has proven a much more challenging task. Second, only less than half of the total Drp1 is found bound to filaments under saturating actin concentration *in vitro* (48). In such conditions, Drp1-actin interactions are influenced by multiple factors, such as ionic strength, GTP/GDP ratio, and Drp1 oligomeric state (42). Third, cellular studies have not detected Drp1 enrichment in actin-rich regions like lamellipodia, filopodia, or stress fibers (42). Instead, Drp1 has been shown to interact with short actin filaments located at the ER-mitochondrial interface (50). Our observations, in line with those of Schulzthaus et al. (43), indicate that LifeAct more predominantly labels long cable F-actin structures, such as the apical actin array and the subapical actin web. As in the case of animal cells, we did not detect any noticeable DnmA enrichment in the areas corresponding to these actin-rich structures. It is possible that at the used resolution, LifeAct may not detect less conspicuous actin filaments.

Overall, there is compelling evidence showing that DnmA homolog Drp1 interacts with actin (36, 48, 50), supporting the idea that specific actin filaments can function as dynamic reservoirs for Drp1 recruitment (42). In animal cells, formin INF2 nucleates a network of short actin filaments at ER-mitochondria contact sites, recruiting Drp1 and allowing the transfer of oligomers to Drp1 receptors, leading to full ring assembly and mitochondrial division (50). Although there are no clear INF2 homologs in *A. nidulans*, the presence of a similar mechanism, counteracting latrunculin and H_2_O_2_ actin depolymerizing activity, might explain why the large DnmA aggregates formed in the absence of FisA remain associated to mitochondria. It is also possible that DnmA larger assemblies interact with exposed mitochondrial cardiolipin, as cardiolipin translocation from the mitochondrial internal membrane to the outer membrane occurs under stress conditions, including mitophagy, and a cardiolipin-binding motif has been identified in Drp1 variable region (51).

The fact that H_2_O_2_ promotes actin depolymerization and reorganization, potentially affecting exocytic and endocytic processes, is interesting in itself. In animal cells, actin filament subunits are directly oxidized at methionine 44, which is conserved from fungi to humans, by oxidoreductases from the MICAL family. This oxidation severs filaments and decreases polymerization (52). MICAL enzymes not only show direct interaction with actin but also produce H_2_O_2_ *in vivo* and *in vitro*, which can also modify cysteine and tryptophane residues, affecting actin polymerization and depolymerization (53). However, there is some controversy regarding whether actin depolymerization is mediated (54) or not (55) by MICAL-produced H_2_O_2_, rather than by direct hydroxylation of actin methionine residues. Since there are no MICAL homologs present in fungi, it would be interesting to determine the mechanisms by which H_2_O_2_ induces actin depolymerization in these organisms. In any case, our results suggest that the redox regulation of actin polymerization is an ancestral mechanism that has been preserved during evolution.

In addition to actin depolymerization, H_2_O_2_ induced an increased production of actin patches that were distributed at the cell cortex as well as in the cytoplasm. These actin patches are associated with endocytic and exocytic activities (56). Such changes could be the result of H_2_O_2_-induced actin depolymerization. However, our results showing that DnmA C450 is critical for DnmA function in mitochondrial and peroxisomal scission (see below) suggest that H_2_O_2_ might affect endocytosis/exocytosis in a more direct way. In fact, DnmA C450 is conserved in human dynamins 1 and 2 as well as in *A. nidulans* dynamin VpsA (AN8023). VpsA homolog Vps1 in *S. cerevisiae* is involved in vacuolar morphology and has a major endosomal function. Like in the case of DnmA, those functions depend on a proper dynamin assembly and GTP hydrolysis (57). *A. nidulans* VpsA has been little studied, but *vpsA* gene disruption results in decreased growth and vacuole fragmentation (58). Therefore, it is possible that H_2_O_2_ might directly affect dynamin membrane scission functions during endocytosis/exocytosis.

In our efforts to understand the effects of H_2_O_2_-mediated oligomerization of DnmA, we mutagenized four conserved cysteines. Of these, C776 is located in the GED domain, which is part of the neck or BSE region. The GED domain is critical for both the assembly of higher-order complexes and the cooperative stimulation of GTPase activity and, therefore, for mitochondrial division (59). Within that region, the mutation of Drp1 strictly conserved K679 (K679A) impaired GTPase activity and increased Drp1 complex levels by either increasing its formation or decreasing its disassembly (60). Consistent with this, the mutation of C776 (C776S), which is highly conserved only in fungi, affected DnmA organization, promoting its aggregation (Fig. 11), affecting development (Fig. 6 and 7), and specifically impairing H_2_O_2_-induced mitochondrial division (Fig. 8 and 11). Notably, charged amino acids around C776 that would affect C776 redox properties are well conserved in fungi (Fig. S4).

The other three cysteines analyzed (C380, C450, and C462) are all located in the DnmA stalk region. However, only C450 was found essential for DnmA function. DnmA C450 corresponds to human Drp1 C431. A patient with Drp1 mutation C431Y died at the age of 10 months, and this variant was unable to restore normal mitochondrial morphology in Drp1 null HTC116 animal cells. However, in contrast to our results, the equivalent mutation in yeast Dnm1 (C466Y) showed only moderate defects in mitochondrial division and exhibited minor dominant negative effects when co-expressed with wild-type Dnm1 (31). Human Drp1 C431 is located in the stalk α2^S^ helix, which is part of Drp1 (also named DNM1L) stalk interface 2 which is crucial for its assembly. Charge reversal mutations at the center of this interface (E490R and K642E) interfere with dimerization and generate monomeric species, while E490A has no effect on the assembly of the protein (61). In sharp contrast, C450S replacement did not eliminate DnmA dimerization, additional oligomerization, or FisA-mediated mitochondrial recruitment, despite eliminating DnmA function. Moreover, in diploid cells, C450S mutant did not behave as a dominant mutation.

Nevertheless, H_2_O_2_-induced formation of DnmA larger assemblies was clearly delayed in the C450S mutant, when compared to wild-type DnmA (Fig. 11; Fig. S7).

In *Dictyostelium discoideum*, the chemical modification of Drp1 homolog dynamin A cysteine residues C375 and C831, induced by the protease inhibitor ZPCK, resulted in the exclusive formation of stable ring-like structures *in vitro* (62). Like DnmA C450 and C776, these cysteine residues are located in the middle and GED domains, respectively, confirming the importance that cysteine residues located in these regions have in oligomerization dynamics and ultimate architecture of the members of this protein family.

Our molecular dynamics results reveal that C450S and C776S substitutions, along with the oxidative modification of C450 (C450-SO2^−^), exert pronounced influences on several facets of DnmA’s structure. These effects encompass alterations in the DnmA BSE-stalk angle, solvent-accessible surface area, and salt bridge interactions. The inverse correlation in protein structural changes found for C450S and C776S substitutions is consistent with the opposite effect that these mutations had in oligomerization in the absence of FisA. Moreover, the high probability of C450 oxidation and its associated changes in DnmA secondary structure, helix bending, and hydrogen bonding interactions indicate that the *in vivo* oxidation of C450 by H_2_O_2_ would impact DnmA oligomeric structure and, therefore, its scission functionality. Here, it is important to underscore that DnmA C450 and C462 equivalent cysteines in *Magnaporthe oryzae* MoDnm1 (MGG_06361) were the only cysteines identified as oxidation sensitive in a global redox proteome analysis (63), further supporting the possibility that C450 can be oxidized *in vivo*.

We found that *A. nidulans* largely filamentous mitochondria are normally highly decorated with oligomeric forms of either wild-type, C450S, or C776S DnmA proteins, bound to FisA. However, this alone is clearly not sufficient to induce mitochondrial division. As indicated, a close ER-mitochondrial contact (11, 47) and the presence of H_2_O_2_ are necessary for extensive mitochondrial division (5, 11). Furthermore, in absence of mitochondrial division caused by the lack of DnmA or FisA, both ER-mitochondrial contacts and H_2_O_2_ are required to produce mitochondrial constrictions (11). This suggests that FisA-recruited DnmA undergoes additional priming events at ER-mitochondrial contact sites to transition into the productive assembly needed for mitochondrial scission at constriction sites. Notably, specific events that can impact the redox status of Drp1 have been described at the ER-mitochondrial interface. One is the role of protein disulfide isomerase as a thiol reductase affecting Drp1 redox status (64), and the other is the presence of specific H_2_O_2_ nanodomains at such interface (65).

Based on this and our results, we propose that H_2_O_2_ produced at ER-mitochondria contact sites (65) coordinates both the generation of mitochondrial constrictions (11) and the priming of recruited DnmA oligomers by regulating the oxidation status of DnmA C450. Mitochondrial constriction and DnmA priming for scission are both necessary for efficient mitochondrial division. A similar priming can be postulated for peroxisomal division, considering that peroxisomes are generated *de novo* by the ER and that mitochondria also contribute to peroxisome biogenesis (66). Also relevant is the fact that peroxisomes generate H_2_O_2_ during fatty acid oxidation and that peroxisome division in yeast is initiated by an internal signal that centers around the repositioning of acyl-CoA oxidase, an H_2_O_2_-producing enzyme, from the matrix to the membrane. In the membrane, this enzyme interacts with peroxisome biogenesis protein Pex16 and initiates phosphatidic acid and diacylglycerol biosynthesis. This lipid synthesis, coupled with the transbilayer relocation of diacylglycerol, allows the assembly of dynamin-related protein Vps1 and actin cytoskeleton proteins to the peroxisomal surface, in a process that culminates in peroxisome division (67).

## MATERIALS AND METHODS

### Strains, media, and growth conditions

*Aspergillus nidulans* strains used in this work are listed in Table S1. All strains were cultivated on supplemented nitrate-minimal medium with 1% glucose or oleate as sole carbon sources (68). Sexual development was induced as reported (45). In brief, 1 × 10^5^ conidia were inoculated using soft agar on plates containing supplemented 2% glucose solid minimal medium. After 24 h, the plates were sealed with masking tape and incubated at 37°C for 8 days.

### Strain construction

The list of primers utilized in this work is listed in Table S2. The construction to replace wild-type *dnmA* (AN8874) gene with a *dnmA::gfp* fusion, a double-joint PCR method, was employed (69). Two PCR fragments were generated using wild-type genomic DNA as template and the following primer pairs: GSP1DnmA/GSP2DnmA and GSP3DnmA/GSP4DnmA. The *Aspergillus fumigatus gfp::pyrG* fragment was amplified from plasmid PFNO3 (70), using primers GFP1DnmA and GFP2DnmA. These three fragments were purified and used in a fusion PCR with nested primers 5′NestDnmA and 3′NesDnmA. The final 6,100-bp DnmA–GFP-AfpyrG cassette was purified and introduced into the *A. nidulans* strain A1155 by electroporation (71, 72). Five transformants were obtained, and four were analyzed by PCR for asexual development and for the presence of the DnmA::GFP protein (Fig. S1), using a Nikon Eclipse E600 epifluorescence microscope, connected to a Neo Andor sCMOS cooled camera.

Point mutations in *dnmA* were also generated by double joint PCR, where the TGC cysteine codon was replaced by the TCT serine codon. For each mutation, two PCR fragments were obtained using genomic DNA from the TVG5 (*dnmA::gfp*) strain and the following primer pairs: GSP1DnmA/C/S295Rev and C/S295For/GSP4DnmA, GSP1DnmA/C/S380Rev and C/S380For/GSP4DnmA, GSP1DnmA/C/S450Rev and C/S450For/GSP4DnmA, GSP1DnmA/C/S462Rev and C/S462For/GSP4DnmA, and GSP1DnmA/C/S776SRev and C/S776For/GSP4DnmA. The fragments were purified, mixed, and used in fusion PCRs with nested primers 5′NestDnmA and 3′NesDnmA. The final 6,100-bp dnmAC/S–GFP-AfpyrG cassettes were purified and used to transform *A. nidulans* strains A1155 and/or CVG8 by electroporation. At least three transformants were obtained in each case, and their *dnmA* alleles were fully sequenced to verify the presence of the desired mutation and the absence of any additional change. Strains TVG5 (*dnmA::gfp*) and TVG6 (*dnmAC776S::gfp*) were crossed to strain TRV1 to label mitochondria and eliminate the *kuA* deletion, resulting in strains CVG14 and CVG33. Strains TVG9 (*dnmAC295S::gfp*), TVG10 (dnmAC380S::gfp), TVG11 (*dnmAC462S::gfp*), and TVG15 (*dnmAC450S::gfp*) were crossed to CLK43 strain to eliminate *kuA* deletion and obtain strains CVG40, CVG41, CVG24, and CVG53.

To obtain strain with labeled peroxisomes, strains TVG5 (*dnmA::gfp*), TVG6 (*dnmAC776S::gfp*), and TVG16 (*dnmAC450S::gfp*) were crossed with strain CDC014 (mcherry::FLAG-PTS1), derived from strain RPA520 (73). The progeny was analyzed using epifluorescence microscopy, and strains CVG59, CVG55, and CVG56 were obtained. Diploid strains DVG1, DVG7, DVG2, DVG6, and DVG4 were generated using the following strain pairs: CVG14 (*dnmA::GFP*)/A1155 (*dnmA*), CVG53 (*dnmAC450S::gfp*)/TVG15 (*dnmAC450S::gfp*), CVG14 (*dnmA::gfp*)/TVG15 (*dnmAC450S::gfp*), CVG53 (*dnmAC450S::gfp*)/A1155 (*dnmA*), and CVG33 (*dnmAC776S::gfp*)/A1155 (*dnmA*).

### Confocal and Airyscan microscopy

Confocal images were acquired using a Zeiss LSM800 inverted laser scanning confocal microscope using a Plan Apochromat 63×/1.4 oil immersion objective. Laser lines at 405, 488, or 561 nm were used according to the fluorescent tag. Airyscan images were obtained with an LSM800 microscope and a Plan-Apochromat 63×/1.4 oil DIC M27 objective, using Airyscan detectors (Carl Zeiss, Jena, Germany), configured with a 32-channel array of GaAsP detectors at 1.25 airy units per channel. All of the observations were conducted at 34°C. For maximal intensity Z projections, six slices were acquired with an interval of 0.7 µm through the entire cell volume (5 µm). The images were processed using Zen 2012 (Carl Zeiss, Jena, Germany) and Fiji package from ImageJ 2 (v2.030/1.53f). For fixed images, the samples were incubated with 300 µL of a 1:100 dilution of stock fixing solution 2.46 M paraformaldehyde, 0.44 M EGTA, 0.5 M MgCl, 100 mM PIPES (pH 6.9) for 3 min, rinsed three times with water, and observed using epifluorescence or confocal microscopy. MitoSOX Red (Invitrogen Waltham, MA, USA) was used to detect mitochondrial ROS as reported (5). A 5 mM stock solution was prepared in DMSO and maintained frozen. This solution was diluted with water to a final 5 µM working concentration. The solution was used to cover sections of solid medium containing growing mycelia for 20 min at 37°C in the dark. Afterward, the MitoSOX Red solution was removed, and the mycelia were rinsed twice with sterile water and immediately observed using confocal microscopy. The confocal microscopy settings used for detection were excitation at 510 nm and emission at 580 nm. Ten hyphal tips were analyzed for each strain, and the fluorescence signal intensity of a fixed area (40 µm) along mitochondria was used to obtained equivalent gray mean values.

### Protein structure analysis

For system preparation, we obtained the DnmA AlphaFold (74, 75) model available at Uniprot (https://www.uniprot.org/uniprotkb/Q5AS56/entry) and removed the variable domain unstructured region from residues 557 to 699 and add caps. PropKa (76) and pCysMod (77) servers were used to predict amino acids pKa and cysteine modifications of the protein. From the starting model, we derived the mutation of C450S and C776S systems and the sulfinylated product of C450 as the cysteine sulfinate C450-SO_2_^−^ system. Topology files were prepared in the Amber18 *tleap* module using the force field of proteins ff14SB (78) and the TIP3P (79) water model. The four systems were solvated in 20 Å of water molecules in all directions, forming a rectangular box. Na^+^ and Cl^−^ ions were added to obtain 0.15 M NaCl.

### MD simulations

First, the energy of the systems was minimized using 1,000 steepest descent steps followed by 1,000 conjugate gradient steps. Full electrostatic forces were treated using the particle mesh Ewald (PME) method (80), and van der Waals interactions were computed using a 10-Å non-bonded cutoff radius. All molecular dynamic simulations were run with a 2-fs time step and constrained hydrogen-containing bonds using SHAKE algorithm (81, 82). Langevin dynamics (83) was used for temperature control with a collision frequency of 2 ps^−1^. The systems were heated by linearly varying the target temperature from 0 to 310 K for 80 ps at constant volume and maintaining it at 310 K for an additional 20 ps, applying cartesian positional restraints to the protein backbone with a force constant of 50 kcal/molÅ^2^. Next, a restricted number of particles, system pressure, and temperature (NPT) equilibration was carried out at 310 K and 1 bar of pressure for 10 ns applying cartesian positional restraints to the backbone atoms with a force constant of 50 kcal/molÅ^2^. The next three NPT equilibrations were performed under the same conditions for 5 ns each with force constants of 25, 10, and 5 kcal/molÅ^2^, consecutively. Then, a restraintless NPT equilibration of 5 ns was carried out, followed by the 500-ns production. In all NPT simulations, Berendsen barostat (84) was used to control pressure with a relaxation time of 1 ps.

### MD data analysis

RMSD, RMSF, SASA, distance secondary structure, and hydrogen bond calculation and analysis were performed using cpptraj (85). Angle calculations were carried out using MDAnalysis (85, 86) and an in-house Python (87) script. Structure images were generated using PyMOL v0.9, and graphs were made using Python. As hydrogen bond criteria, we used a cutoff distance of 3 Å between the donor (D) and acceptor (A) atoms and an angle of at least 135° (∠DHA. cpptraj used the DSSP [88] algorithm for secondary structure analysis and calculated the SASA using LCPO [89]).

## SUPPLEMENTAL MATERIAL

Supplemental material mbio.02822-23-s0001.pdf Figures S1-S11 and movie captions.

Supplemental Tables mbio.02822-23-s0002.docx Tables S1-S4.

Movie S1 mbio.02822-23-s0003.mp4 DnmA forms highly dynamic different-size oligomers mostly associated to dividing and non-dividing mitochondria, with some oligomers localized in the cytoplasm.

Movie S2 mbio.02822-23-s0004.mp4 In the absence of its receptor FisA, H_2_O_2_ induces the formation of large dynamic DnmA assemblies that remain associated to mitochondria.

ASM does not own the copyrights to Supplemental Material that may be linked to, or accessed through, an article. The authors have granted ASM a non-exclusive, world-wide license to publish the Supplemental Material files. Please contact the corresponding author directly for reuse.
