# Supplementary material for: Filamentous actin destabilization by H2O2 favors DnmA aggregation, with crucial roles of cysteines 450 and 776 in mitochondrial and peroxisomal division in Aspergillus nidulans
Source: mBio. 2023 Nov 28;14(6):e02822-23. doi: 10.1128/mbio.02822-23 (PMC10746283; doi:10.1128/mbio.02822-23)
Supplement: Supplemental material — Figures S1-S11 and movie captions. [file mbio.02822-23-s0001.pdf]

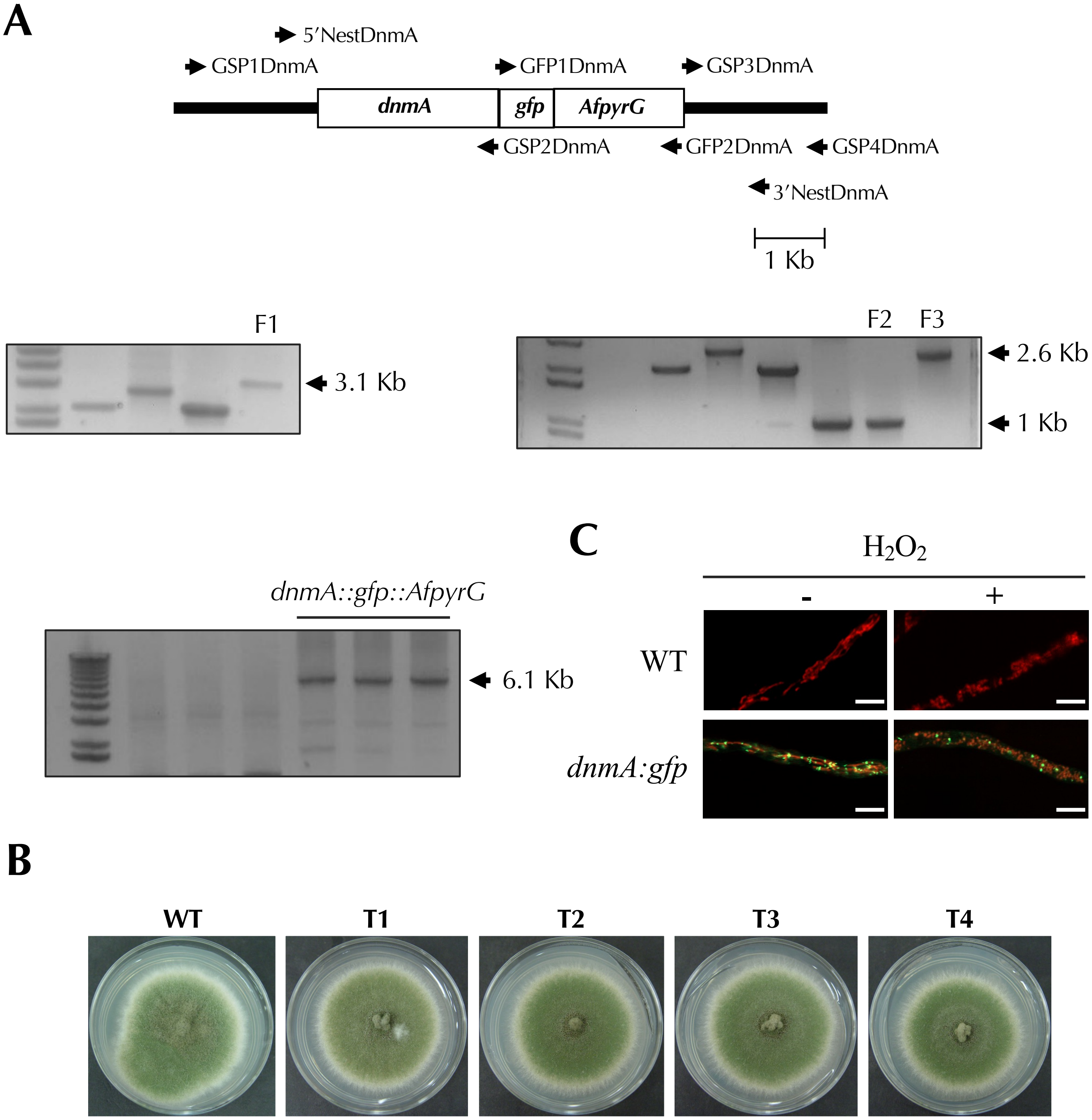

FIG S1 Generation of strains expressing DnmA::GFP from the *dnmA* promoter. A. *dnmA* gene 5′ and 3′ PCR fragments were obtained with primers GSP1DnmA/GSP2DnmA (F1) and GSP3DnmA/GSP4DnmA (F2), respectively. GFP-AfpyrG was amplified using plasmid PFNO3 as a template and primers GFP1DnmA/GFP2DnmA (F3). A final *dnmA::gfp::AfpyrG* 6.1 Kb PCR product obtained with primers 5′NESTDnmA and 3′NESTDnmA was purified and used to transform strain A1155. **B**. Growth and conidiation patterns of recipient strain A1155 (WT) and four of the transformants obtained. Transformant T1 was named TVG5 and used in further experiments **C**. Strains TRV1 (WT, with mCherry labelled mitochondria) and TVG5 (*dnmA::gfp,* stained with Mitotracker red) were treated or not with 5 mM H_2O_2 for 20 minutes and observed using confocal microscopy. Mitochondria and DnmA::GFP signals are shown in red and green, respectively. The scale bars represent 5 μm.

MOVIE S1 DnmA forms highly dynamic different-size oligomers mostly associated to dividing and non-dividing mitochondria, with some oligomers localized in the cytoplasm. A growing hyphae from strain CVG14 was recorded for the indicated time. The mitochondrial matrix was labelled with mts::mCherry and DnmA was labelled with GFP. Arrowhead points to a cytoplasmic oligomer that shows anterograde and retrograde movements.

MOVIE S2 In the absence of its receptor FisA, H_2O_2 induces the formation of large dynamic DnmA assemblies that remain associated to mitochondria. Mycelia from strain CVG24 (Δ*fisA dnmA::gfp*) grown for 22 h were treated or not with 5 mM H_2O_2 for 20 min. H_2O_2 was removed and hyphae were recorded for the indicated time. The mitochondrial matrix was labelled with mts::mCherry and DnmA with GFP.

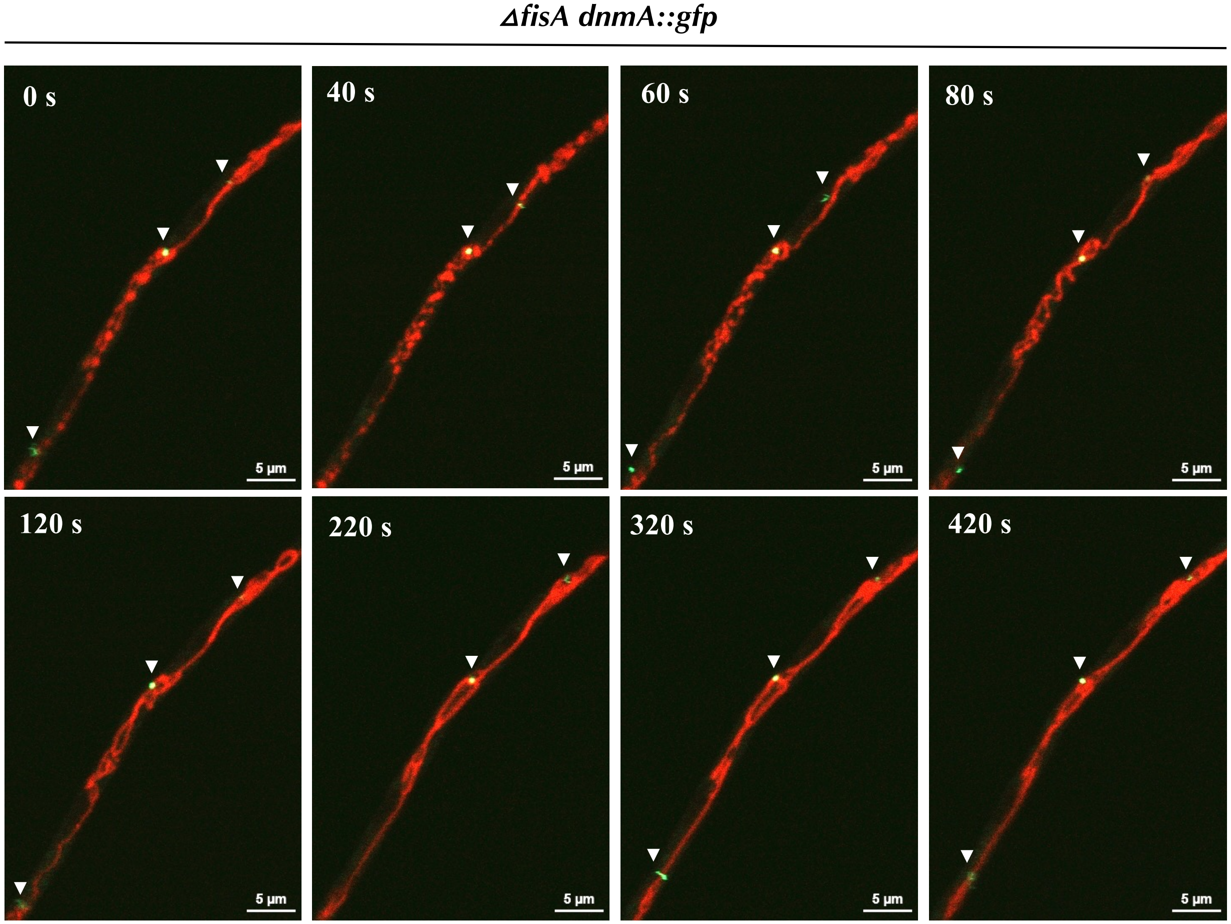

FIG S2 In the absence of its receptor FisA, DnmA is dispersed throughout the cytoplasm, where H_2O_2 induces the formation of large and dynamic DnmA assemblies that remain associated to mitochondria. Mycelia from strain CVG24 (Δ*fisA dnmA::gfp*) grown for 22 h were treated with 5 mM H_2O_2 for 20 min. H_2O_2 was removed and hyphae were observed at the indicated times (seconds) using Airyscan microscopy. The mitochondrial matrix was labelled with mts::mCherry and DnmA with GFP. DnmA::GFP assemblies are indicated by arrowheads. Scale bar represents 5µm.

H_2O

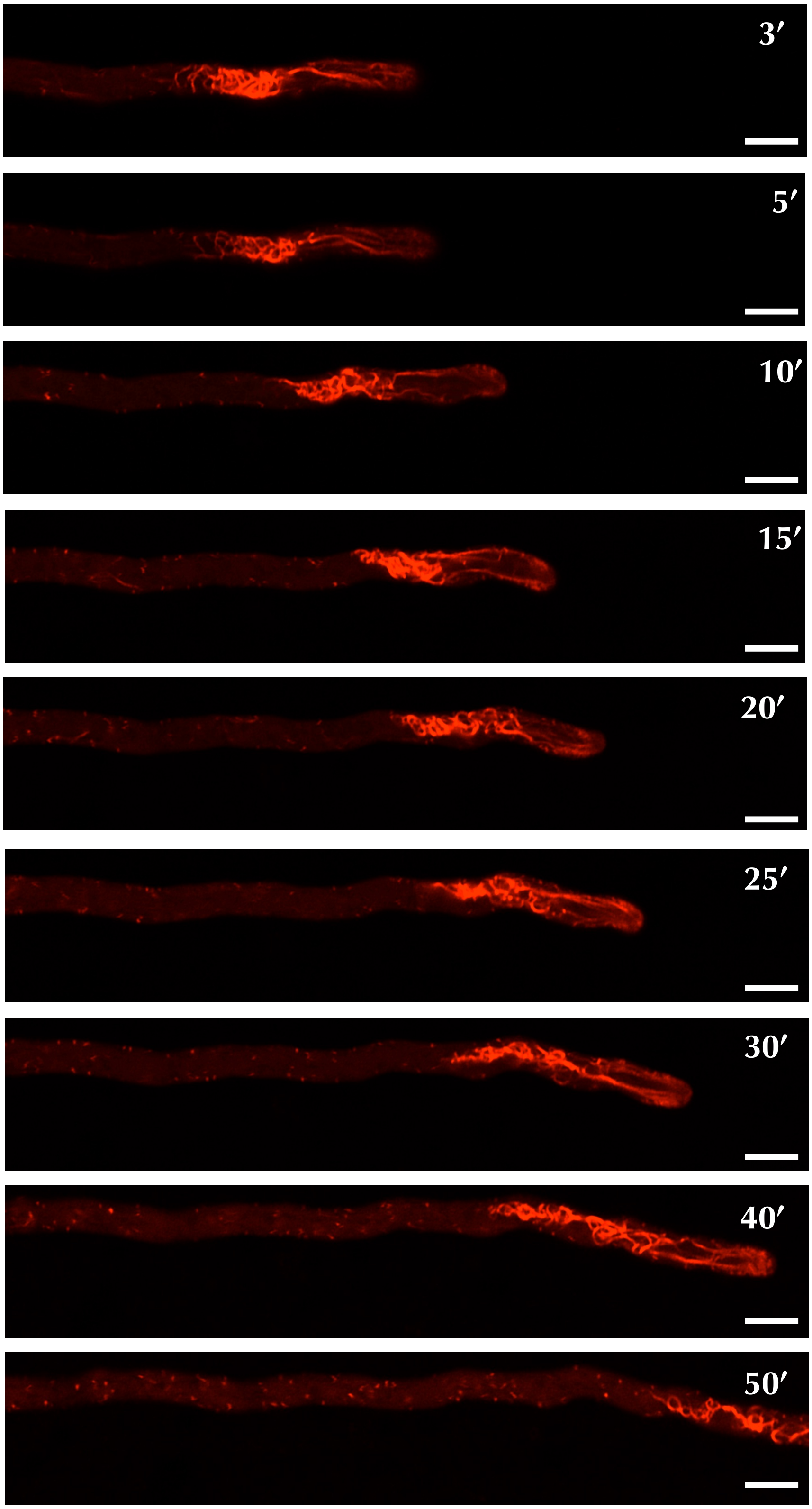

FIG S3. During hyphal growth the subapical actin network undergoes continuous reshaping, indicating actin polymerization and depolymerization events. Mycelia from strain LQR3 (*LifeAct::TagRFP*) grown for 22 h were incubated with water instead of H_2O_2 or latrunculin, and after two minutes continuous *in vivo* observations were made using Airyscan microscopy. Pictures were taken at the specified time intervals. The subapical actin web or SAW is observed in red. The scale bar represents 5 μm.

A

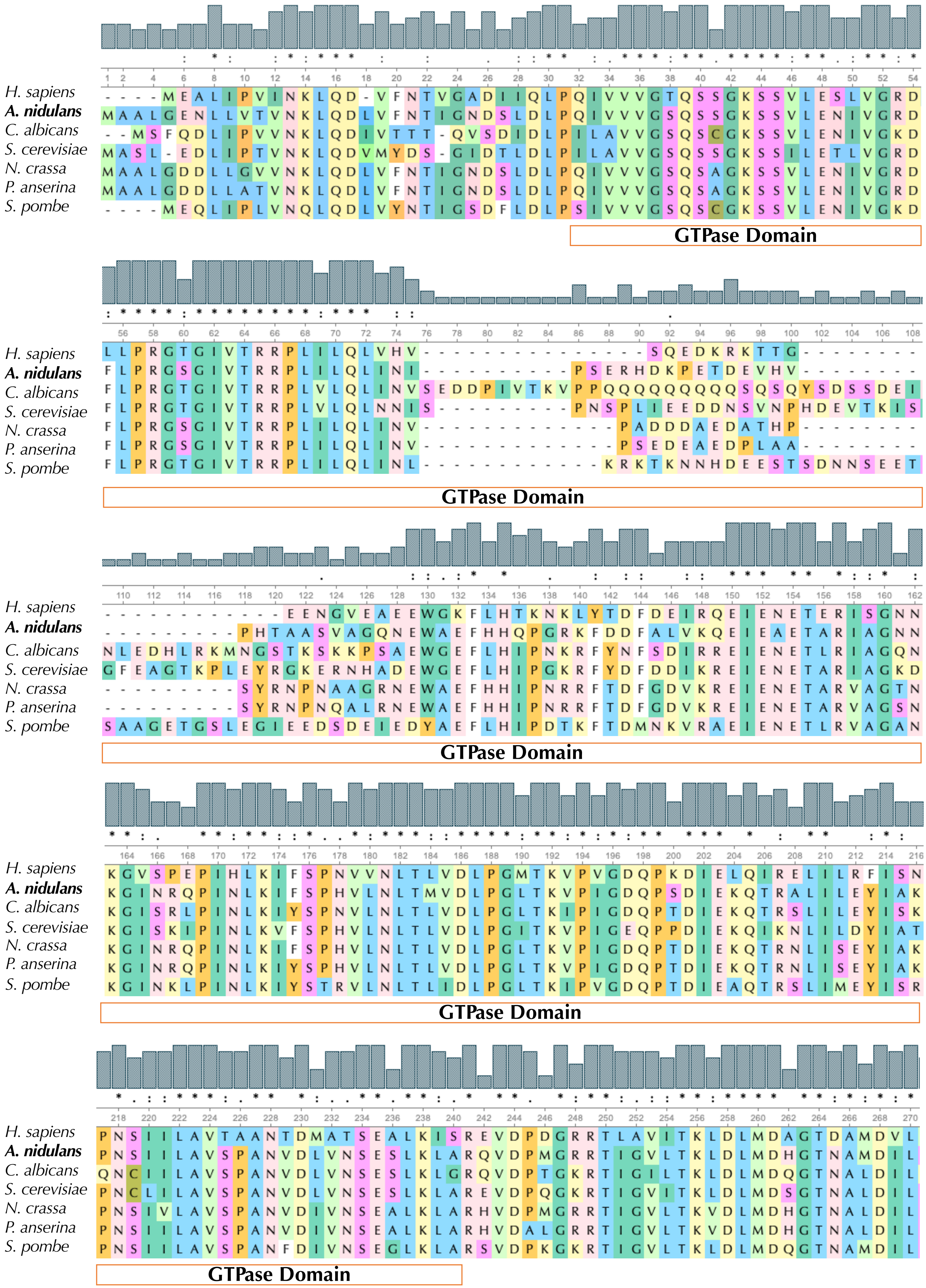
H. sapiens
A. nidulans
C. albicans
S. cerevisiae
N. crassa
P. anserina
S. pombe
GTPase Domain

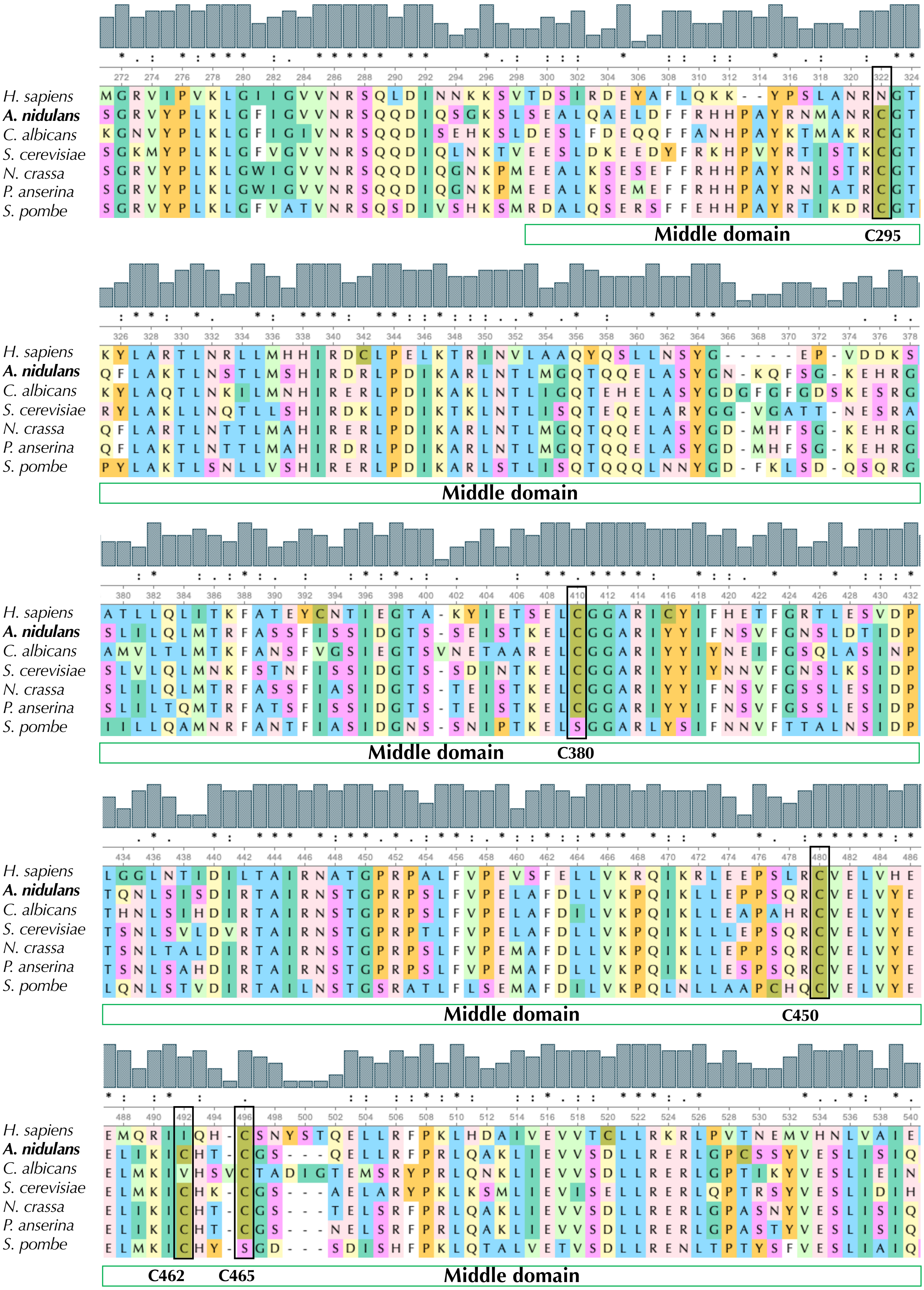
H. sapiens
A. nidulans
C. albicans
S. cerevisiae
N. crassa
P. anserina
S. pombe
Middle domain
C295
C380
C450
C462
C465

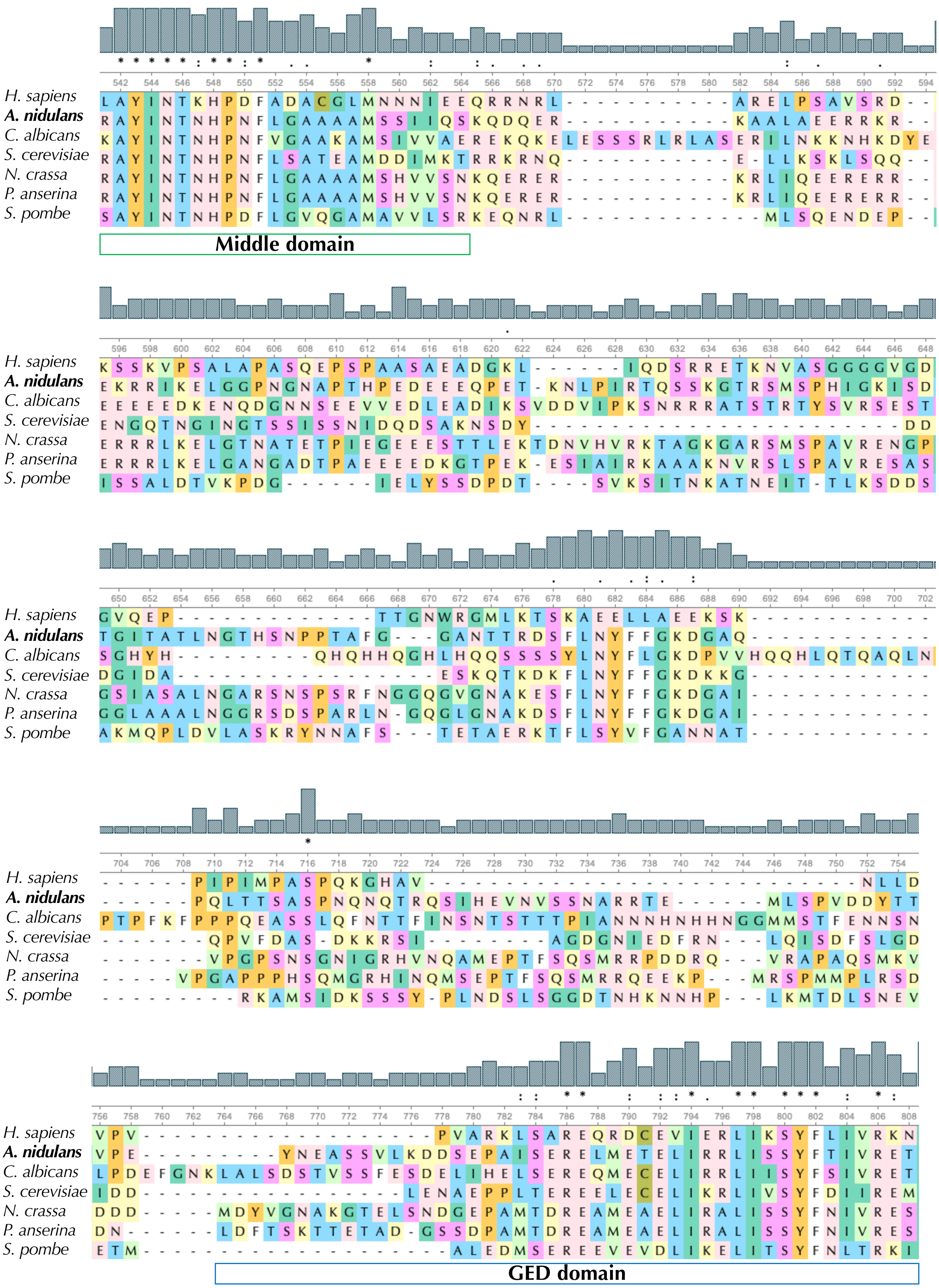
H. sapiens LAYINTKHPDFADACGLMNNNIEEQRRNRL-------------ARELPSAVSRD--
A. nidulans RAYINTNHPNFLGAAAAMSSIIQSKQDQER-------------KAALAEERRKR--
C. albicans KAYINTNHPNFVGAAKAMSIVVAEREKQKELESSSRLRLASERILNKKNHKDYE
S. cerevisiae RAYINTNHPNFLSATEAMDDIMKTRRKRNQ-------------E-LLKSKLSQQ--
N. crassa RAYINTNHPNFLGAAAAMSHVVSNKQERER-------------KRLIQEERERR--
P. anserina RAYINTNHPNFLGAAAAMSHVVSNKQERER-------------KRLIQEERERR--
S. pombe SAYINTNHPDFLGVQGAMAVVLSRKEQNRL--------------MLSQENDEP--
Middle domain
H. sapiens KSSKVPSALAPASQEPSPAASAEADGKL------IQDSRRETKNVASGGGGVGD
A. nidulans EKRRIKELGGPNGNAPTHPEDEEEQPET-KNLPIRTQSSKGTRSMSPHIGKISD
C. albicans EEEEEDKENQDGNNSEEVVEDLEADIKSVDDVIPKSNRRRATSTRTYSVRSEST
S. cerevisiae ENGQTNGINGTSSISSNIDQDSAKNSDY------------------------DD
N. crassa ERRRLKELGTNATETPIEGEEESTTLEKTDNVHVRKTAGKGARSMSPAVRENGP
P. anserina ERRRLKELGANGADTPAEEEEDKGTPEK-ESIAIRKAAAKNVRSLSPAVRESAS
S. pombe ISSALDTVKPDG------IELYSSDPDT----SVKSITNKATNEIT-TLKSDDS
H. sapiens GVQEP-------------TTGNWRGMLKTSKAEELLAEEKSK-----------
A. nidulans TGITATLNGTHSNPPTAFG---GANTTRDSFLNYFFGKDGAQ-----------
C. albicans SGHYH---------QHQHHQGHLHQQSSSSYLNYFLGKDPVVHQQHLQTQAQLN
S. cerevisiae DGIDA-----------------ESKQTKDKFLNYFFGKDKKG-----------
N. crassa GSIASALNGARSNSPSRFNGGQGVGNAKESFLNYFFGKDGAI-----------
P. anserina GGLAAALNGGRSDSPARLN-GQGLGNAKDSFLNYFFGKDGAI-----------
S. pombe AKMQPLDVLASKRYNNAFS---TETAERKTFLSYVFGANNAT-----------
H. sapiens ------PIPIMPASPQKGHAV------------------------------NLLD
A. nidulans ------PQLTTSASPNQNQTRQSIHEVNVSSNARRTE------MLSPVDDYTT
C. albicans PTPFKFPPPQEASSLQFNTTFINSNTSTTTPIANNNHNHHNGGMMSTFENNSN
S. cerevisiae -------QPVFDAS-DKKRSI--------AGDGNIEDFRN---LQISDFSLGD
N. crassa -------VPGPSNSGNIGRHVNQAMEPTFSQSMRRPDDRQ---VRAPAQSMKV
P. anserina -----VPGAPPPHSQMGRHINQMSEPTFSQSMRRQEEKP---MRSPMMPLRSD
S. pombe ---------RKAMSIDKSSSY-PLNDSLSGGDTNHKNNHP---LKMTDLSNEV
H. sapiens VPV-----------------PVARKLSAREQRDCEVIERLIKSYFLIVRKN
A. nidulans VPE---------YNEASSVLKDDSEPAISERELMETELIRRLISSYFTIVRET
C. albicans LPDEFGNKLALSDSTVSSFESDELIHELSEREQMECELIRRLIISYFSIVRET
S. cerevisiae IDD----------------LENAEPPLTEREELECELIKRLIVSYFDIIREM
N. crassa DDD-----MDYVGNAKGTELSNDGEPAMTDREAMEAELIRALISSYFNIVRES
P. anserina DN------LDFTSKTTETAD-GSSDPAMTDREAMEAELIRALISSYFNIVRES
S. pombe ETM-----------------ALEDMSEREEVEVDLIKELITSYFNLTRKI
GED domain

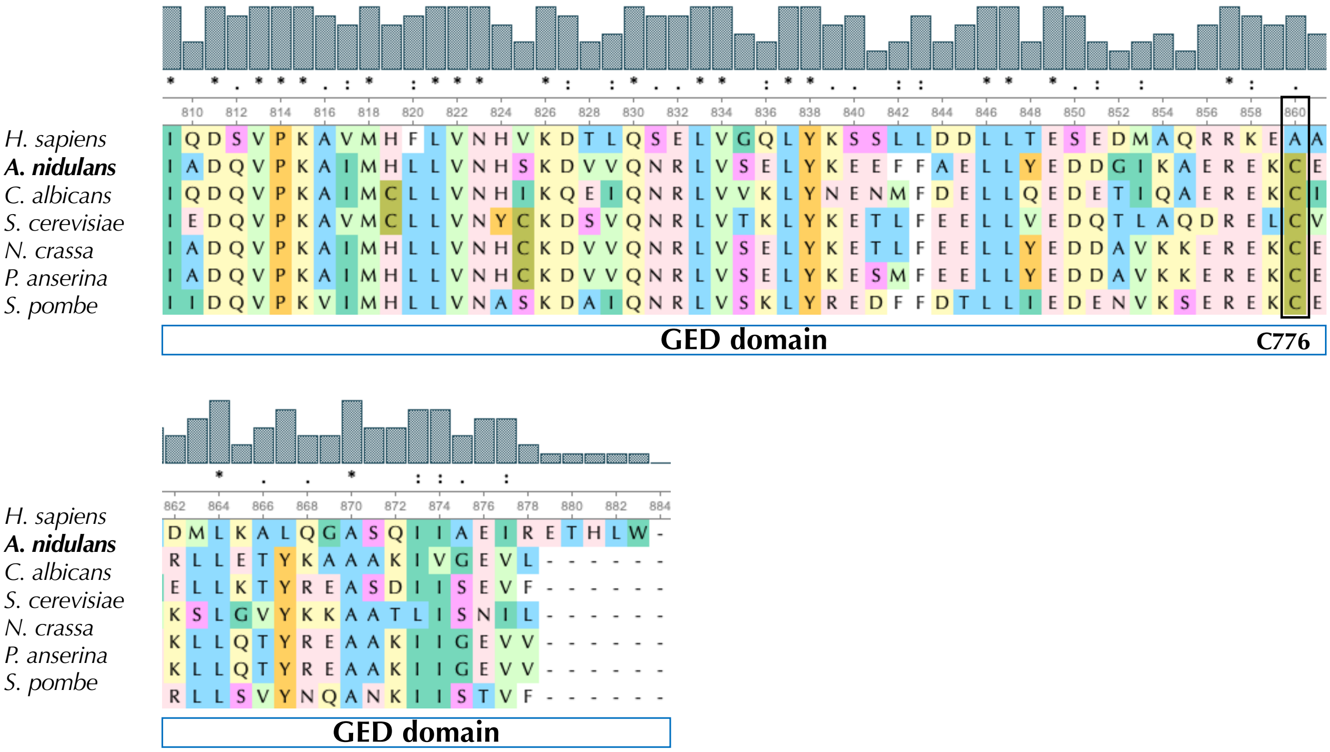

FIG S4 DnmA is a member of the highly conserved dynamin-relared protein family. *A. nidulans* DnmA (AN8874) was aligned to the corresponding homologs from *Homo sapiens* and the indicated fungi. Uniprot accession numbers are *Homo sapiens,* 00429; *Aspergillus nidulans,* A0A1U8QLZ1; *Candida albicans,* A0A1D8PKC9; *Saccharomyces cerevisiae,* P54861; *Neurospora crassa,* Q7SDJ3; *Podospora anserina,* Q2WCN9; and *Schizosaccharomyces pombe* Q09748. All characteristic domains and conseved cysteines are indicated.

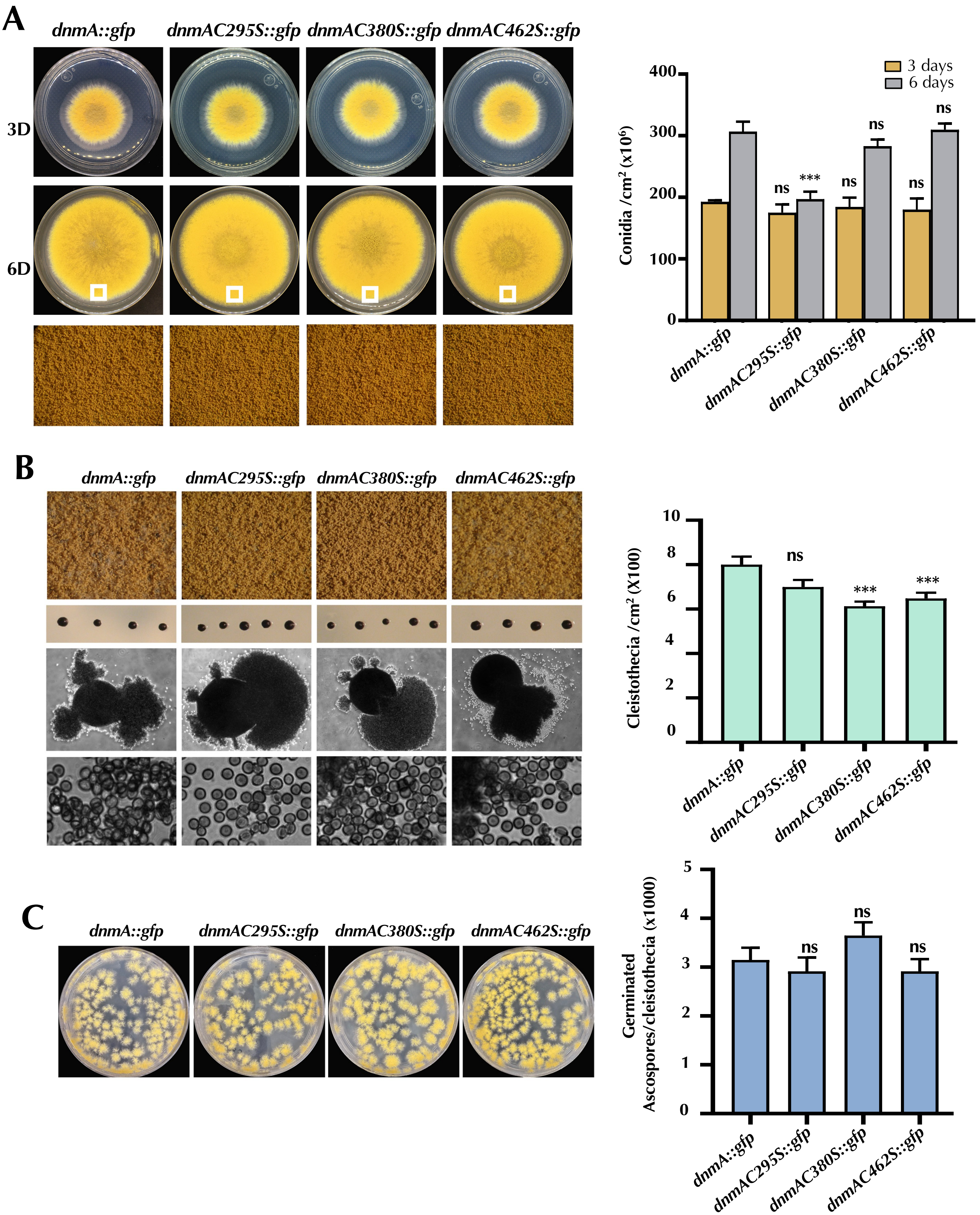

FIG S5 DnmA C295S, C380S, and C462S substitutions produced minor or no effects on growth, asexual (A) and sexual development (B-C). Strains CVG14 (*dnmA::gfp*), CVG40 (*dnmAC295S::gfp*), CVG41 (*dnmAC380S::gfp*) and CVG42 (*dnmAC462S::gfp*) were analyzed as in **Figs 5-6**. Yellow and gray bars represent the number of conidia per square centimeter for 3D and 6D colonies, respectively. The standard deviation from three independent experiments is shown. Green and blue bars represent the number of cleistothecia and germinated ascospores produced by each strain. Data was analyzed by one-way ANOVA, followed by Dunnet's test ($*p< 0.05$). Asterisks and ns indicate significant and no significant differences with respect to the control strain CVG14 (*dnmA::gfp*).

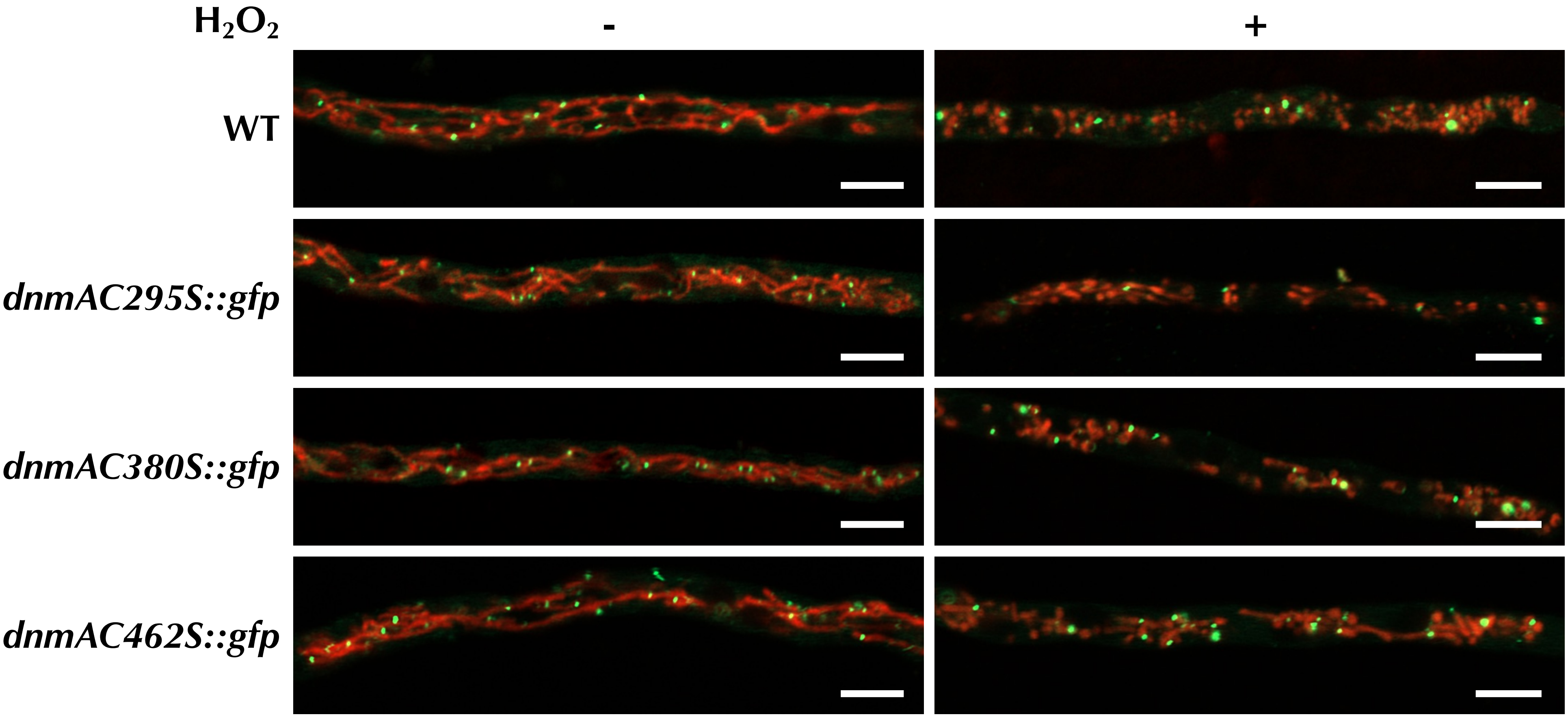

FIG S6 DnmA C295S, C380S, and C462S substitutions do not affect H_2O_2-induced mitochondrial division. Mycelia from strains Strains CVG14 (*dnmA::gfp*), CVG40 (*dnmAC295S::gfp*), CVG41 (*dnmAC380S::gfp*) and CVG42 (*dnmAC462S::gfp*) grown for 22 h, were treated or not with 5 mM H_2O_2 for 20 min. H_2O_2 was removed and mycelia were observed using Airyscan microscopy. The scale bar represents 5 μm.

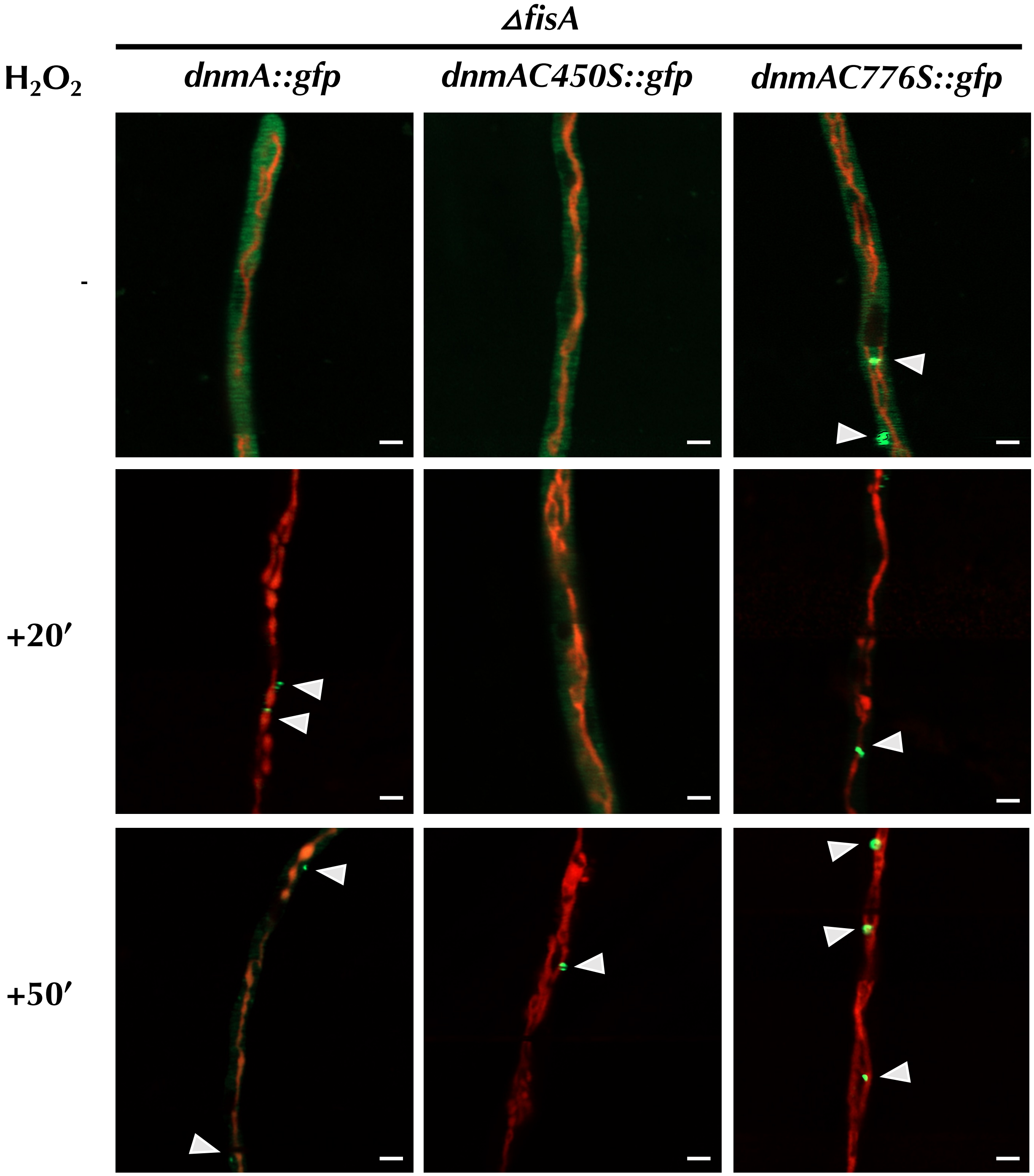

FIG S7 In absence of FisA, DnmA C450S and C776S substitutions result in delayed and premature DnmA oligomerization, respectively. Mycelia from strains CVG24 (Δ*fisA dnmA::gfp*), CVG58 (Δ*fisA dnmAC450S::gfp*), and CVG43 (Δ*fisA dnmAC776S::gfp*) were grown for 22 h and treated with or without 5 mM H_2O_2 for the indicated time (min). After removing H_2O_2, hyphae were observed using Airyscan microscopy. DnmA Oligomers are indicated by white arrowheads. The scale bar represents 2 μm.

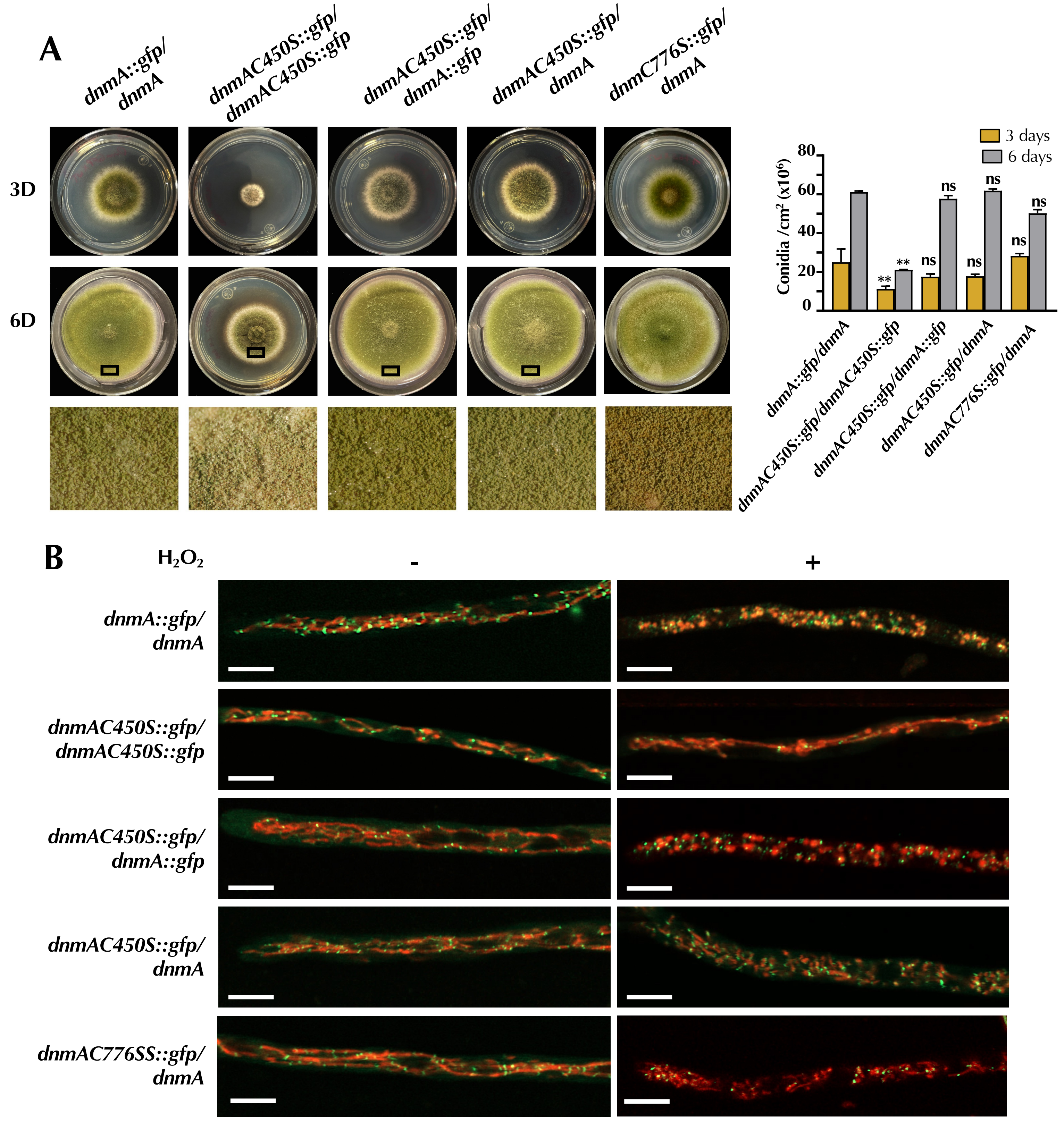

FIG S8 Mutations *dnmAC450S* and *dnmAC776S* do not behave as dominant negative. A. Conidia ($1x10^4$) from diploid strains DVG1 (*dnmA::GFP/dnmA*), DVG7 (*dnmAC450S::gfp/dnmAC450S::gfp*), DVG2 (*dnmAC450S::gfp /dnmA::gfp*), DVG6 (*dnmAC450S::gfp/dnmA*), and DVG4 (*dnmAC776S::gfp/dnmA*) were inoculated on minimal medium plates and incubated at 37°C for 3 (3D) or 6 (6D) days. The lower panel shows an enlarged view of the colony edges (indicated by black squares). The total number of conidia per colony area was quantified (right panel). The yellow and gray bars represent the number of conidia per square centimeter for 3D and 6D colonies, respectively. The standard deviation from three independent experiments is shown. Data was analyzed by one-wayANOVA, followed by Dunnet's test (*$p< 0.05$). Asterisks indicate significant differences with respect to the control strain DVG1. **B**. The same diploid strains were grown for 22 h and treated with or without 5 mM H_2O_2 for 20 min. After removing H_2O_2, hyphae were observed using Airyscan microscopy. The scale bar represents 5µm.

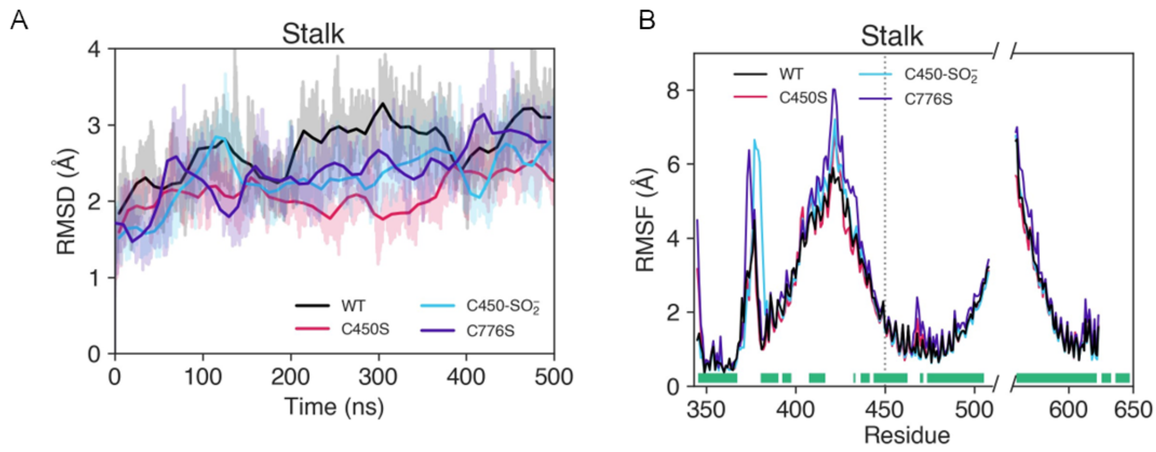

Figure S9. **A**. Backbone root mean square deviation. **B**. Residue root mean square fluctuation of the stalk for each simulated system.

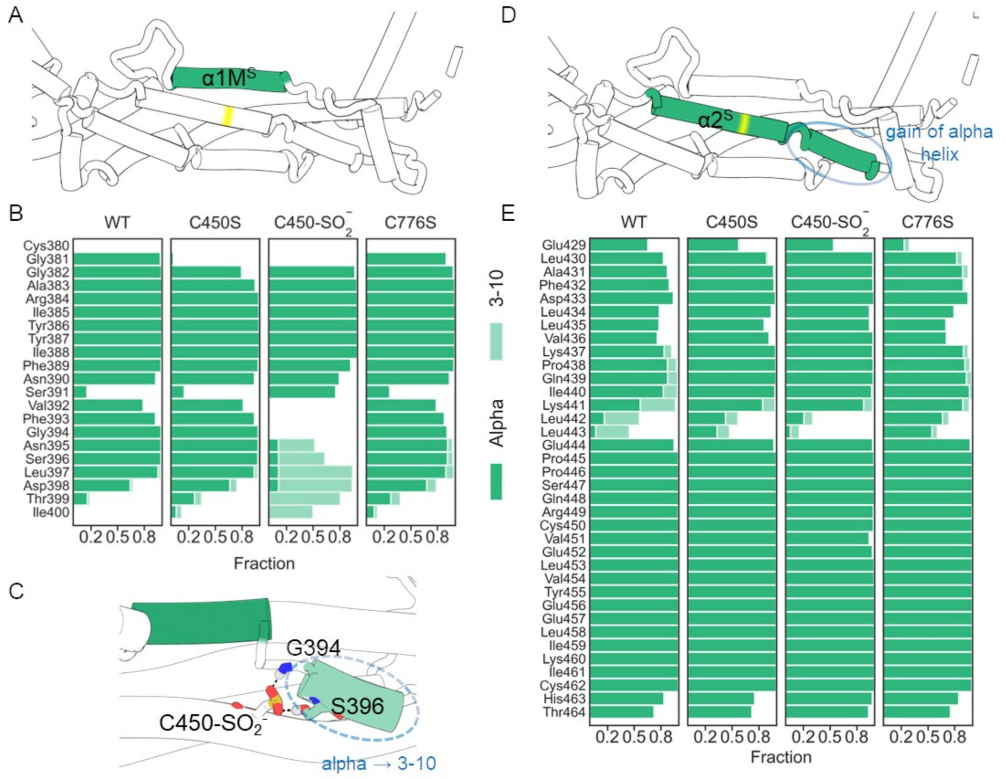

FIG S10. Predicted changes in DnmA stalk and BSE domain secondary structure when C450 is oxidized. A. 3D representation of the WT stalk. **B**. SS fraction of the α1M^S helix. **C**. Hydrogen bond representation between C450-SO_2^- and α1M^S residues. **D**. 3D representation of the stalk. **E**. SS fraction of the α2^S helix.

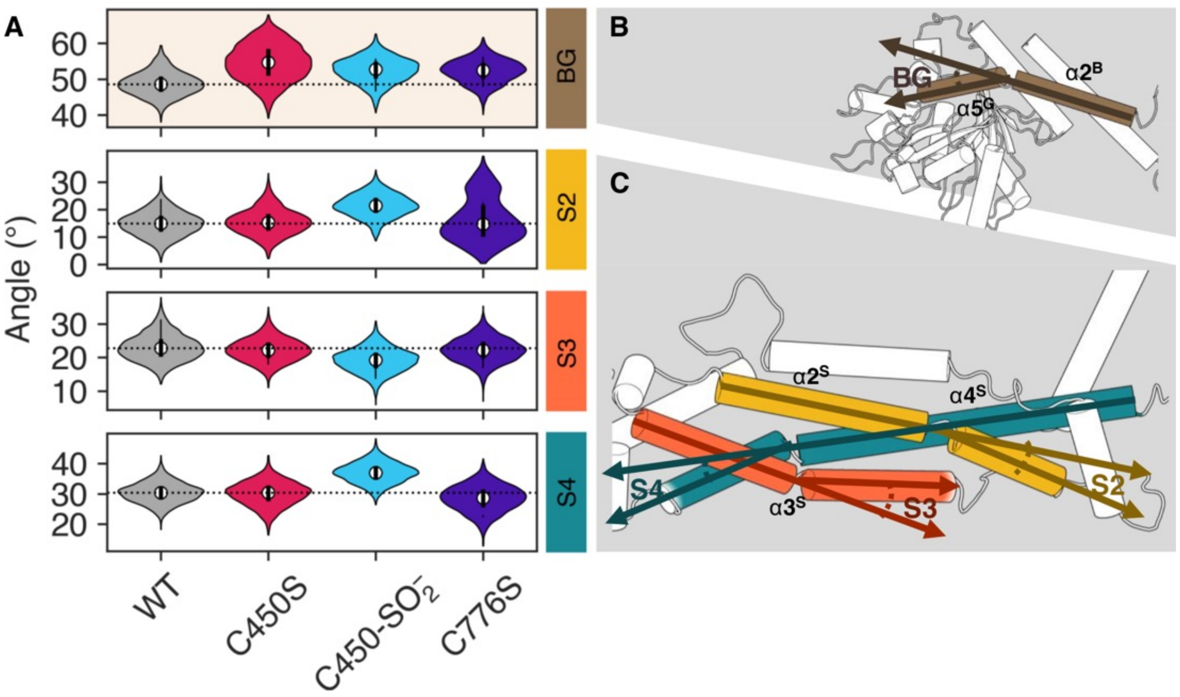

FIG S11. Bending of some DnmA helices is altered by C450S, C450-SO2⁻, and C776S changes. A. Distributions of bending angles of the different helices in each of the simulated systems. **B**. Representation of each measured angle.
