# Supplementary material for: Filamentous actin destabilization by H2O2 favors DnmA aggregation, with crucial roles of cysteines 450 and 776 in mitochondrial and peroxisomal division in Aspergillus nidulans
Source: mBio. 2023 Nov 28;14(6):e02822-23. doi: 10.1128/mbio.02822-23 (PMC10746283; doi:10.1128/mbio.02822-23)
Supplement: Supplemental Tables — Tables S1-S4. [file mbio.02822-23-s0002.docx]

**Table S1**. *Aspergillus nidulans* strains used in this work

| Strain | Genotype | Source |
| --- | --- | --- |
| CLK43 | *pabaA1 yA2 veA1* | Kawasaki *et al*., (2002) |
| A1155 | *pyrG89 pyroA4 ∆nkuA::bar veA1* | Fungal Genetics Stock Center |
| A26 | *biA veA1* | Fungal Genetics Stock Center |
| LQR3 | *pyrG89::niiA(p)::LifeAct::TagRFP; pyroA4; wA3; veA1; AfpyrG* | Schultzhaus *et al*., 2016 |
| TRV1 | *pabaA1 yA2 gpdA (p)::atp9-7(mts)::mcherry veA1* | Garrido-Bazán *et al*., (2020) |
| TVG2 | *pyrG89 pyroA4 ΔfisA::AfpyrG pyroA4 ΔnkuA::bar veA1^a^* | Garrido-Bazán *et al*., (2020) |
| TVG5 | *pyrG89 pyroA4 dnmA::gfp::AfpyrG ∆nkuA::bar veA1^a^* | This work; A1155 transformed with PCR construct dnmA-gfp-AfpyrG |
| TVG7 | *pyrG89 pyroA4 dnmAC776S::gfp::AfpyrG pyroA4 ΔnkuA::bar veA1^a^* | This work; A1155 transformed with PCR construct dnmAC776S-gfp-AfpyrG |
| TVG9 | *pyrG89 pyroA4 dnmAC295S::gfp::AfpyrG ΔnkuA::argB gpdA (p)::atp9-7(mts)::mcherry veA1^b^* | This work; CVG8 transformed with dnmAC295S-gfp-AfpyrG |
| TVG10 | *pyrG89 pyroA4 dnmAC380S::gfp::AfpyrG ΔnkuA::argB gpdA (p)::atp9-7(mts)::mcherryveA1^b^* | This work; CVG8 transformed with dnmAC380S-gfp-AfpyrG |
| TVG11 | *pyrG89 pyroA4 dnmAC462S::gfp::AfpyrG ΔnkuA::argB gpdA (p)::atp9-7(mts)::mcherry veA1^b^* | This work; CVG8 transformed with dnmAC462S-gfp-AfpyrG |
| TVG15 | *pyrG89 pyroA4 dnmAC450S::gfp::AfpyrG ΔnkuA::argB gpdA (p)::atp9-7(mts)::mcherry veA1^b^* | This work; CVG8 transformed with dnmAC450S-gfp-AfpyrG |
| TVG16 | *pyrG89 pyroA4 dnmAC450S::gfp::AfpyrG pyroA4 ΔnkuA::bar veA1^a^* | This work; A1155 transformed with PCR construct dnmAC450S-gfp-AfpyrG |
| CVG1 | *pabaA1 yA2 ∆dnmA::AfpyrG gpdA (p)::atp9-7(mts)::mcherry veA1^a^* | Garrido-Bazán *et al*., (2020) |
| CVG2 | *pabaA1 yA2 ∆fisA::AfpyrG gpdA (p)::atp9-7(mts)::mcherry veA1^a^* | Garrido-Bazán *et al*., (2020) |
| CVG8 | *pyrG89 pyroA4 gpdA (p)::atp9-7(mts)::mcherry ∆nkuA::argB veA1^b^* | Garrido-Bazán *et al*., (2022) |
| CVG14 | *pabaA1 yA2 dnmA::gfp::AfpyrG gpdA (p)::atp9-7(mts)::mcherry veA1^a^* | This work; progeny from TVG5 x TRV1 |
| CVG24 | *pabaA1 yA2 ΔfisA::AfpyrG dnmA::gfp::AfpyrG gpdA (p)::atp9-7(mts)::mcherry veA1^a^* | This work; progeny from CVG14 x TVG2 |
| CVG30 | *pabaA1 yA2 ∆dnmA::AfpyrG yA::[gpdA(p)-mcherry-FLAG-PTS1::Afpyro] veA1* | Garrido-Bazán *et al*., (2020) |
| CVG31 | *pabaA1 yA2 ∆fisA::AfpyrG yA::[gpdA(p)-mcherry-FLAG-PTS1::Afpyro] veA1* | Garrido-Bazán *et al*., (2020) |
| CVG33 | *pabaA1 yA2 dnmAC776S::gfp::AfpyrG gpdA (p)::atp9-7(mts)::mcherry veA1^a^* | This work; progeny from TVG7 X TRV1 |
| CVG35 | *pabaA1 yA2 dnmA:gfp::AfpyrG veA1^a^* | This work: progeny from TVG5 x TRV1 |
| CVG40 | *pabaA1 yA2 dnmAC295S::gfp::AfpyrG gpdA (p)::atp9-7(mts)::mcherry veA1^a,b^* | This work: progeny from TVG9 X CLK43 |
| CVG41 | *pabaA1 yA2 dnmAC380S::gfp::AfpyrG gpdA (p)::atp9-7(mts)::mcherry veA1 ^a,b^* | This work: progeny from TVG10 X CLK43 |
| CVG42 | *pabaA1 yA2 dnmAC462S::gfp::AfpyrG gpdA (p)::atp9-7(mts)::mcherry veA1 ^a,b^* | This work: progeny from TVG11 X CLK43 |
| CVG43 | *pabaA1 yA2 ΔfisA::AfpyrG dnmC776S::gfp::AfpyrG gpdA (p)::atp9-7(mts)::mcherry veA1^a^* | This work; progeny from TVG2 X CVG33 |
| CVG53 | *pabaA1 yA2 dnmAC450S::gfp::AfpyrG gpdA (p)::atp9-7(mts)::mcherry veA1^a^* | This work; progeny from TVG5 X CLK43 |
| CVG55 | *pabaA1 yA2 dnmAC450S::gfp::AfpyrG yA:: [gpdA(p)-mcherry-FLAG-PTS1::Afpyro] veA1* | This work: progeny from CDC14x TVG16 |
| CVG56 | *pabaA1 yA2 dnmAC776S::gfp::AfpyrG yA:: [gpdA(p)-mcherry-FLAG-PTS1::Afpyro] veA1* | This work: progeny from CDC14x TVG7 |
| CVG58 | *pabaA1 yA2 ΔfisA::AfpyrG dnmC450S::gfp::AfpyrG gpdA (p)::atp9-7(mts)::mcherry veA1^a^* | This work; progeny from TVG15 X CVG2 |
| CVG59 | *pabaA1 yA2 dnmA::gfp::AfpyrG yA:: [gpdA(p)-mcherry-FLAG-PTS1::Afpyro] veA1* | This work: progeny from CDC14 X CVG35 |
| CDC14 | *pabaA1 yA::[gpdA(p)-mcherry-FLAG-PTS1::Afpyro] veA1* | This work: progeny from CVG31 X A26 |
| DVG1 | *pyrG+/ pyrG89 yA+/yA2 pyroA+/pyroA4 nkuA+/∆nkuA::bar veA+/veA1 pabaA+/pabaA1 dnmA+/dnmA::gfp::AfpyrG gpdA (p)::atp9-7(mts)::mcherry^a^* | This work: diploid obtained from A1155 X CVG14 |
| DVG2 | *pyrG+/ pyrG89 yA+/yA2 pyroA+/pyroA4 nkuA+/∆nkuA::bar veA+/veA1 pabaA+/pabaA1 dnmA::gfp/dnmAC450S::gfp::AfpyrG gpdA (p)::atp9-7(mts)::mcherry^a^* | This work: diploid obtained from TVG15 X CVG14 |
| DVG4 | *pyrG+/ pyrG89 yA+/yA2 pyroA+/pyroA4 nkuA+/∆nkuA::bar veA+/veA1 pabaA+/pabaA1 dnmA::gfp/dnmAC776S::gfp::AfpyrG gpdA (p)::atp9-7(mts)::mcherry^a^* | This work; diploid obtained from A1155 X CVG33 |
| DVG6 | *pyrG+/ pyrG89 yA+/yA2 pyroA+/pyroA4 nkuA+/∆nkuA::bar veA+/veA1 pabaA+/pabaA1 dnmA+/dnmAC450S::gfp::AfpyrG gpdA (p)::atp9-7(mts)::mcherry^a^* | This work; diploid obtained from A1155 X CVG53 |
| DVG7 | *pyrG+/ pyrG89 yA+/yA2 pyroA+/pyroA4 nkuA+/∆nkuA::bar veA+/veA1 pabaA+/pabaA1 dnmAC450S::gfp::AfpyrG/dnmAC450S::gfp::AfpyrG gpdA (p)::atp9-7(mts)::mcherry^a^* | This work; diploid obtained from TVG15 X CVG53 |

^a^It may contain pyrG89

^b^ It may contain riboB2

**Table S2**. Primers used in this work

| Primer | Sequence (5′ - 3′) |
| --- | --- |
| GSP1DnmA | CAGGCTGCTAGAAGCTACCAAAGGC |
| GSP2DnmA | CAAAACTTCGCCGACAATTTTCGC |
| GFP1DnmA | GAAAATTGTCGGCGAAGTTTTGGGAGCTGGTGCAGGCGCTGG |
| GFP2DnmA | CGGACAGACAGGCGTTATGTCTGTCTGAGAGGAGGCACTGATG |
| GSP3DnmA | ACATAACGCCTGTCTGTCCGTGTT |
| GSP4DnmA | CAGATGCGCAGGCGAAAGG |
| 5’NestDnmA | TGACGAGCTCTGCGGAACGAT |
| 3’NestDnmA | GCGGAGTGCATCTTCTCTCCG |
| C/S 295For | GGCCAATCGATCTGGGACGCAAT |
| C/S 295Rev | ATTGCGTCCCAGATCGATTGGCC |
| C/S 380For | CCACGAAAGAATTATCTGGTGGTGCTAGGATAT |
| C/S 380Rev | ATATCCTAGCACCACCAGATAATTCTTTCGTGG |
| C/S 450For | CTAGCCAGCGGTCTGTTGAGCTG |
| C/S 450Rev | CAGCTCAACAGACCGCTGGCTAG |
| C/S 462For | GAAGAACTTATAAAGATATCTCACACTTGTGGCTCGCA |
| C/S 462Rev | TGCGAGCCACAAGTGTGAGATATCTTTATAAGTTCTTC |
| C/S 776For | CCGAGAGAGAGAAGTCTGAGAGGTTGTTAGAA |
| C/S 776Rev | TTCTAACAACCTCTCAGACTTCTCTCTCTCGG |

**Table S3**. Predicted pKa values for DnmA cysteine residues obtained with PropKa Server.

| Residue | pKa | Localization |
| --- | --- | --- |
| C295 | 9.51 | Buried |
| C380 | 5.37 | Surface |
| C450 | 10.33 | Buried |
| C462 | 12.46 | Buried |
| C465 | 10.51 | Buried |
| C495 | 9.78 | Buried |
| C776 | 9.00 | Surface |

**Table S4**. Predicted modifications of DnmA cysteine residues obtained with pCysMod Server.

| Residue | Modification | % FPR |
| --- | --- | --- |
| C295 | S-sulfenylation | 2.85 |
| C380 | S-sulfinylation | 7.64 |
| C450 | S-sulfinylation | 0.56 |
| C465 | S-sulfinylation | 6.76 |
| C776 | S-sulfenylation | 4.41 |
| C776 | S-sulfinylation | 5.02 |
